# A Survey on an Energy-Efficient and Energy-Balanced Routing Protocol for Wireless Sensor Networks

**DOI:** 10.3390/s17051084

**Published:** 2017-05-10

**Authors:** Olayinka O. Ogundile, Attahiru S. Alfa

**Affiliations:** 1 Department of Electrical, Electronic and Computer Engineering, University of Pretoria, Hatfield 0028, South Africa; attahiru.alfa@umanitoba.ca; 2 Electrical and Computer Engineering, University of Manitoba, Winnipeg, MB R3T 2N2, Canada

**Keywords:** clustering, energy-balanced, energy-efficient, load-balanced tree, multi-hop, multipath, routing protocols, single-hop, single-path, WSNs

## Abstract

Wireless sensor networks (WSNs) form an important part of industrial application. There has been growing interest in the potential use of WSNs in applications such as environment monitoring, disaster management, health care monitoring, intelligence surveillance and defence reconnaissance. In these applications, the sensor nodes (SNs) are envisaged to be deployed in sizeable numbers in an outlying area, and it is quite difficult to replace these SNs after complete deployment in many scenarios. Therefore, as SNs are predominantly battery powered devices, the energy consumption of the nodes must be properly managed in order to prolong the network lifetime and functionality to a rational time. Different energy-efficient and energy-balanced routing protocols have been proposed in literature over the years. The energy-efficient routing protocols strive to increase the network lifetime by minimizing the energy consumption in each SN. On the other hand, the energy-balanced routing protocols protract the network lifetime by uniformly balancing the energy consumption among the nodes in the network. There have been various survey papers put forward by researchers to review the performance and classify the different energy-efficient routing protocols for WSNs. However, there seems to be no clear survey emphasizing the importance, concepts, and principles of load-balanced energy routing protocols for WSNs. In this paper, we provide a clear picture of both the energy-efficient and energy-balanced routing protocols for WSNs. More importantly, this paper presents an extensive survey of the different state-of-the-art energy-efficient and energy-balanced routing protocols. A taxonomy is introduced in this paper to classify the surveyed energy-efficient and energy-balanced routing protocols based on their proposed mode of communication towards the base station (BS). In addition, we classified these routing protocols based on the solution types or algorithms, and the input decision variables defined in the routing algorithm. The strengths and weaknesses of the choice of the decision variables used in the design of these energy-efficient and energy-balanced routing protocols are emphasised. Finally, we suggest possible research directions in order to optimize the energy consumption in sensor networks.

## 1. Introduction

The progress in modern technologies has motivated the design of small electronic low-powered sensor devices. Ordinarily, a considerable number of these sensors are deployed in remote areas in the form of a wireless network of nodes to measure different physical values. This kind of network scenarios are referred to as wireless sensor networks (WSNs). WSNs are serviceable in numerous industrial applications. For instance, WSNs find use in environment monitoring and disaster management applications such as forest fire detection, landslide detection, and air pollution detection [1,2,3,4]. In intelligent surveillance and defence reconnaissance, WSNs are used in applications such as distributed situation awareness and geographic directed queries [5,6]. Also, WSNs have been extensively used in health care monitoring activities such as mass-casualty disaster, cancer detection and blood glucose measurement [7,8,9]. In these and many other applications, WSNs technology provides different advantages (such as ease of implementation, lower implementation cost, accuracy, scalability) in comparison to the traditional networking solutions [10].

**Figure 1:**
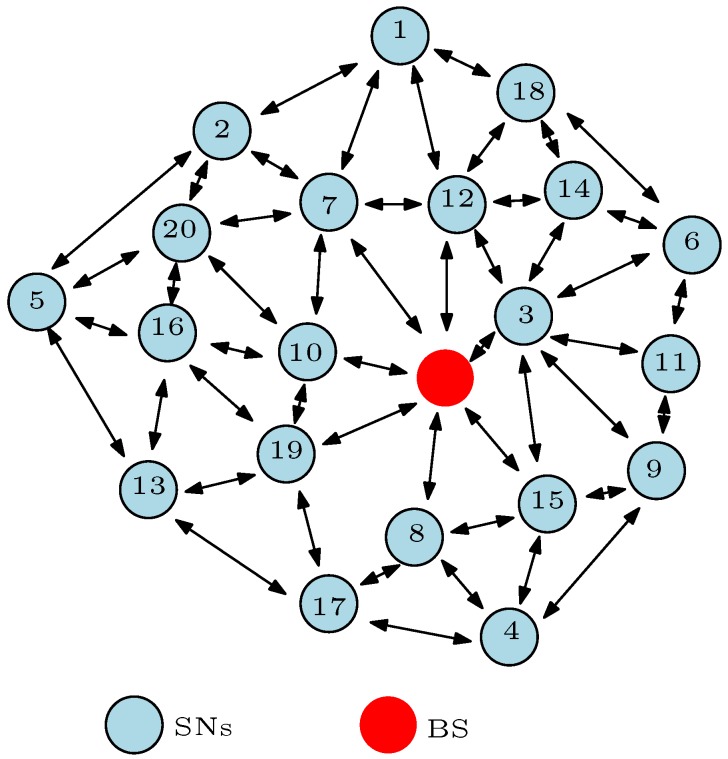
Simplified diagram of a wireless sensor network (WSN).

WSNs conventionally consist of hundreds of sensor nodes (SNs) that communicate with each other in order to convey high quality information to the base station (BS) without using pre-existing infrastructure. This means that WSNs are deployed in an ad hoc manner and the SNs are self-organizing. The SNs are randomly placed in a remote area or sensor field whereby they form a connection with each other in order to measure a physical value as shown in Figure 1. Besides, it is very difficult to replace these SNs after complete deployment in many scenarios or applications, especially in harsh environments such as mines, battlefields, etc. In most cases, the larger the number of SNs deployed to monitor a geographical area or an event, the more reliable and accurate is the delivered message [11]. In Figure 1, each SN transmits the sensed information to the BS depending on the routing protocol or algorithm used. The routing protocol states how the SNs communicate with each other in order to circulate the sensed information towards the BS and it allows the SNs to select the most cost-efficient route(s) to the BS. The route(s) selection choice is specified by the routing algorithm which is mostly designed in the form of a clustering routing algorithm [12,13,14,15] or a load-balanced tree routing algorithm [16,17,18,19]. A cost-efficient routing protocol is developed to balance as much as possible some features such as scalability, timeliness, and robustness. Most importantly, the routing protocol must minimise the energy consumption so as to extend the network lifetime for a reasonable period. As a result, routing in WSNs is quite challenging because the routing protocol must guarantee a balance in some or all of these Quality of Service (QoS) requirements.

**Figure 2:**
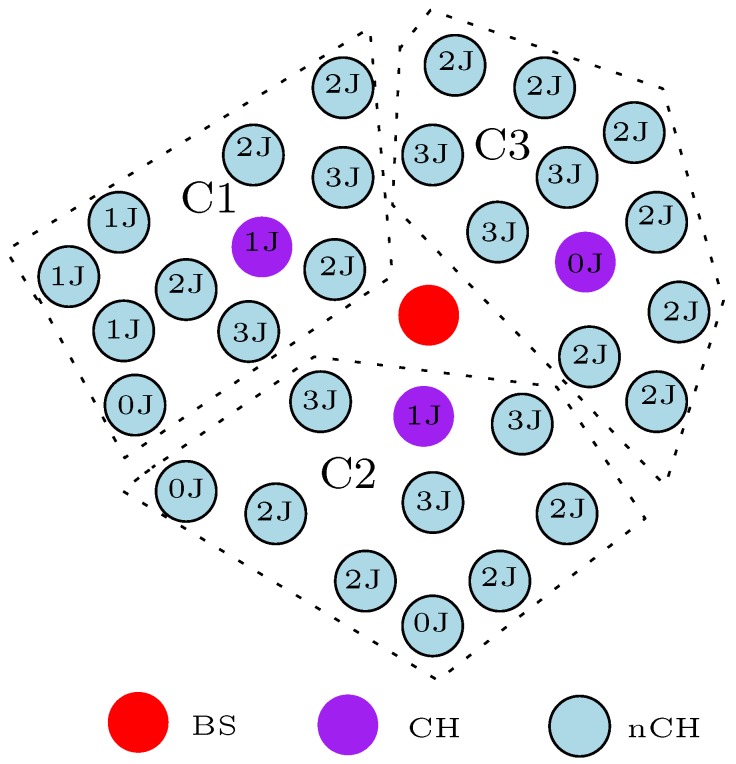
Simplified diagram of a clustered WSN.

With attention on energy consumption, SNs are primarily battery powered devices. The energy consumption of the SN must therefore be properly managed in order to extend the network lifetime and functionality for a reasonable duration, especially if this is all dependent on energy consumption. Different energy-efficient and energy-balanced routing protocols have been proposed for WSNs. The energy-efficient routing protocols attempt to increase the network lifetime by minimizing the energy consumption in each SN. Energy-efficient routing protocol can easily result in untimely partitioning of the sensor network despite there being enough residual energy left in most of the SNs. For example, consider the literal clustering WSN of Figure 2. In this example, the clustering routing algorithm assumes that the cluster head (CH) duty is static, and all non-cluster head (nCH) nodes convey a message to the BS via their CH with single-hop communication. Also, the “*x*” J (J-Joules) in the circles of Figure 2 represents the the energy level (residual energy) of the SNs. As depicted in the figure, the nCH nodes in cluster 3 (C3) still have enough energy to perform another data transmission round or phase but the CH battery energy has been depleted. Hence, all SNs in that cluster are segregated from the whole network and cannot transmit information to the BS. In turn, this debilitates the network functionality.

The energy-balanced routing protocols, on the other hand, protract the network lifetime by uniformly balancing the energy consumption among the SNs in the network. Energy-balanced routing protocols may offer better performance in terms of energy efficiency because their load balances the energy usage among SNs. Therefore, it prolongs the network lifetime and functionality. Although an energy-balanced routing protocol is desirable, designing an optimal energy-balanced routing protocol for WSNs can be a NP-hard problem because of the network structure and formulations. Some of the “energy-balanced routing protocols” in literature may be energy-efficient but do not—in the actual sense—offer an optimal load-balanced feature during the network data transmission phase. As such, using these routing protocols in a large-scale WSN scenario often result in untimely partitioning of the network.

The energy-efficient and energy-balanced routing protocols in the literature have been proposed using different solution types or algorithms such as heuristic, meta-heuristic, linear programming (LP), evolutionary approach, game theory, and swarm intelligence. The focus of most of these routing protocols has been to optimize the energy consumption during the network data transmission activities. Although, a lot of energy is gobbled by the SN components, even while in inactive mode, this survey emphasised only on the routing protocols developed to optimize the energy consumption during the network data transmission phase. Also, this survey only considers routing towards the BS. Consequently, we present a systematic investigation of the different state-of-the-art energy-efficient and energy-balanced routing protocols. The advantages and disadvantages of these energy-efficient and energy-balanced routing protocols are analysed. A taxonomy is introduced to classify the energy-efficient and energy-balanced routing protocols surveyed in this paper based on their proposed mode of communication towards the BS. Moreover, there are factors or parameters to be considered when designing an energy-efficient or energy-balanced routing protocol for WSNs. As such, this paper classifies these routing protocols based on the input decision variables, stating the advantages and disadvantages of using these design decision variables. Besides, we classified the surveyed energy-efficient and energy-balanced routing protocols based on the solution types or algorithms.

The contribution and relevance of this paper is as follows. We note that there have been various survey papers put forward by researchers to review the performance and classify the different energy-efficient routing protocols for WSNs as discussed in Section 2. However, there seems to be no clear survey emphasizing the importance, concepts, and principles of load-balanced energy routing protocols for WSNs. This survey paper provides a clear picture of both the energy-efficient and energy-balanced routing protocols for WSNs. Hence, we present an extensive survey of the different state-of-the-art energy-efficient and energy-balanced routing protocols that attempt to optimize the energy consumption in SNs so as to prolong the network lifetime. With this in mind, the paper introduces a taxonomy to classify the surveyed routing protocols based on their mode of communication towards the BS and the technique(s) used in their implementation. Moreover, we grouped these surveyed routing protocols based on the solution types or algorithms, and the decision variables used in the routing algorithm. Additionally, the strengths and weaknesses of the decision variables used in the design of these routing protocols are discussed. Finally, this survey presents possible research directions in order to optimize the energy consumption in sensor networks. We expect that this survey will help researchers and practitioners in this field to perceive the different energy-efficient and energy-balanced routing techniques in order to make decisions based on their application requirements and network formulations. Although, recent research work such as wireless rechargeable sensor networks (WRSNs) has emerged as an alternative in solving the battery problems associated with the traditional battery powered WSN, we emphasise that this survey focuses purely on battery powered SNs. For reference on WRSNs, refer to [20,21,22,23,24,25,26,27,28,29].

The rest of this paper is structured as follows. Section 2 presents the related survey work on routing protocols for WSNs. The terms and parameters used in this paper are defined in Section 3. In Section 4, the WSN energy model is explained. We explain, using a typical WSN scenario, the concept of energy consumption in SNs during the data transmission and reception phase in this section. Also, the section explains the objectives, principles, and challenges of designing an energy-efficient and energy-balanced routing protocol which forms the basis of our classification. We introduced a taxonomy to classify the studied energy-efficient and energy-balanced routing protocols for WSNs based on their proposed mode of communication towards the BS in Section 5. The categories included in this taxonomy are explained in this section, while also classifying the studied routing protocols under these categories. Also in this section, the surveyed energy-efficient and energy-balanced routing protocols are also classified based on the solution types or algorithms, and the input decision variables used in designing the routing algorithm. In addition, the strengths and weaknesses of the choice of the decision variables used in the studied routing protocols are discussed in this section. In Section 6, we discussed the findings of this survey and presented the possible research directions. Lastly, Section 7 concludes the paper with discernible remarks.

## 2. Related Work

The importance of developing energy-efficient and energy-balanced routing protocols for WSNs cannot be overemphasised. There are numerous ongoing works on the design of energy-efficient and energy-balanced routing protocols for WSNs. Some of these routing protocols may be designed based on the application requirements and the formulation of the network. In addition, the techniques and design requirements of these routing protocols may differ but the ultimate goal is to optimize the energy consumption during the network activities. There are different survey papers on routing protocols in WSNs, and WSNs in general. Nevertheless, we strive to discuss some of the existing survey literature on WSNs and provided the differences between this paper and the existing survey papers.

An extensive survey on the concept of WSNs is presented in [30,31]. The authors discussed in detail the potential applications and factors affecting the design of WSNs. They defined the communication architecture for WSNs, where they surveyed the routing protocols developed for each communication layer. The authors summarise their survey with general possible research directions for WSNs. Quite the contrary, we focus our attention on energy consumption issues with WSNs. This paper provides a taxonomy to classify the energy-efficient and energy-balanced routing protocols for WSNs based on their mode of communication towards the BS. Moreover, the surveyed energy-efficient and energy-balanced routing protocols are classified based on the solution types or algorithms, and the decision variable defined in the routing algorithms.

A general survey on routing techniques in WSNs is provided in [11]. The authors analysed the difficulties in designing a routing protocol for WSNs. Afterwards, the authors classify routing strategies into flat, hierarchical, and location-based routing. This classification is based on the network formulations. They also classified these routing protocols based on other metrics such as negotiation-based, QoS-based, multipath-based, etc. They presented a detailed comparison of these routing algorithms, stating their strengths and weaknesses in terms of the energy and communication overhead savings. Nonetheless, we focus our attention on energy consumption optimization problems. An extensive review of the different state-of-the-art energy-efficient and energy-balanced routing protocols is presented in this paper. More importantly, the taxonomy introduced in this paper is based on the mode of communication used in the energy-efficient and energy-balanced routing technique to convey the sensed message from the source node to the BS, and the decision variables deployed in the design of these studied routing algorithms.

The authors in [32] presented a survey on routing protocols in WSNs. The work in [32] classifies the surveyed routing protocols into three categories: data-centric, hierarchical, and location-based. Their survey work entails routing generally in WSNs, whereby some other QoS requirements are considered. We focus our attention on minimising the energy consumption by SNs in a WSN so as to prolong the network lifetime. In this regard, we surveyed different energy-efficient and energy-balanced routing protocols, discussing their pros and cons. Moreover, this paper classifies these routing protocols based on the solution types or algorithms, and the decision variables defined in the routing algorithm. This paper further discussed possible research directions in order to extend the network lifetime and functionality of WSNs.

In [33], the authors provided a general survey on WSNs. Following a stepwise approach, the authors gave a general overview of numerous WSN applications and review literature on different aspect of WSNs. They described the challenges with WSNs and classified these challenges into three categories, namely: (1) Internal platform and underlying operating system; (2) Communication protocol stack; and (3) Network services, provisioning, and deployment. Furthermore, the authors presented the main research development found in literature on these mentioned categories. Conversely, this paper is aimed at providing directions to researchers for optimizing the energy consumption by SNs in a WSN. We review different literature on energy-efficient and energy-balanced routing protocols for WSNs. As such, we classified the surveyed energy-efficient and energy-balanced routing protocols based on their mode of communication towards the BS, the solution types or algorithms, and the design variables used in each of the studied routing algorithms.

The authors in [34] provided a comprehensive survey on energy conservation in WSNs. Their survey work concentrates on the energy consumed by the SN hardware components. Firstly the authors broke down the energy consumption for the components of a classic SN and divided the SN into four major components, namely: sensing subsystems, processing subsystem, radio subsystem, and power supply unit. In addition, the authors introduced a systematic and extensive taxonomy through which they classified the energy saving schemes into duty-cycling, data-driven, and mobility-based. The mobility-based energy saving was introduced in [34] as a novel energy saving scheme in order to extend the network lifetime of SNs. They further emphasised the importance of conserving the energy consumed by the SNs component, as in the case of the energy consumed during the network data transmission phase. However, as mentioned earlier in Section 1, the emphasis of this paper is on optimizing the energy consumption by SNs during the network data transmission phase, rather than the energy consumed by SN hardware components. Therefore, our discussion is based on the energy-efficient and energy-balanced routing protocols for WSNs. We classified these surveyed routing protocols and created a possible research direction for researchers and practitioners in this area.

A survey on energy-efficient routing protocols for wireless multimedia sensor networks (WMSNs) is provided in [35]. The authors compared the twenty-five surveyed papers based on their performance issues. Additionally, the authors defined the design challenges and limitations of energy-efficient routing protocols for WMSNs. The authors finally classified the surveyed energy-efficient routing protocols based on some metric such as QoS requirement, data delivery model, type of multimedia data, etc. Although the survey in [35] looked at energy-efficient routing protocols, it is only based on routing protocols for WMSNs. In this paper, we concentrate on both energy-efficient and energy-balanced routing protocols and emphasise the importance of a reliable energy-balanced routing protocol.

In [36], an exhaustive survey on routing protocols for WSNs developed based on the swarm intelligence approach is provided. An overview of the principles and applications of swarm intelligence-based routing in WSNs is discussed. The authors proposed a new taxonomy for swarm intelligence-based routing protocols in WSNs. Accordingly, they classified the surveyed swarm intelligence routing protocols based on this taxonomy. They explained the challenges with the use of the swarm intelligence technique for routing in WSNs, thereby identifying the future areas of research. On the other hand, we surveyed swarm intelligence-based energy-efficient and energy-balanced routing protocols for WSNs. We classified these swarm intelligence-based energy-efficient and energy-balanced routing protocols in order to provide direction for interested readers. Moreover, we grouped these swarm intelligence-based energy-efficient and energy-balanced routing protocols according to the defined input decision variables used in the algorithm.

A comprehensive survey is provided in [4,37] on energy-efficient routing protocols for WSNs. The paper in [37] provides a survey on energy-efficient routing protocols for WSNs based on the three categories (data-centric, hierarchical and location-based routing) outlined in [32]. The authors summarised the seventeen surveyed routing techniques based on these categories, stating their strengths and weaknesses. Furthermore, the paper explains the areas of application of the surveyed routing protocols and concluded with open research directions on energy consumption issues with WSNs. The authors in [4] classified these routing protocols based on the topology, communication model, network structure, and reliable routing schemes. Their work also discuss the pros and cons of different energy-efficient routing protocols whereby they presented a comparison among these routing techniques using some measures such as mobility, scalability, power usage, etc. On the other hand, this paper focuses on both energy-efficient and energy-balanced routing protocols. Accordingly, the taxonomy introduced in this paper covers both energy-efficient routing protocols and energy-balanced routing protocols, where we grouped the studied routing protocols based on their mode of communication towards the BS. We also attempt to classify these routing protocols based on the decision variables (hop-distance, residual energy, average network energy) used in achieving optimal energy consumption. Furthermore, this paper grouped the surveyed routing protocols based on the solution types or algorithms in order to enable researchers to make decisions based on their network applications and scenarios.

The survey in [10] gives a general overview of WSNs, stating the areas of application and challenges of WSNs. The authors reviewed the prime research work and testbeds, standards and platforms, and the techniques and principles of WSNs. Besides, they outlined the current happenings in WSN research that considers the possible interaction between WSNs and other technologies such as mobile robots, micro-blog, sensorcloud, etc. The authors explained how this synergy will assist WSNs to realise the right potential. Their survey is concluded with open research directions for interested researchers. Nevertheless, we narrowed down our survey to energy consumption issues with WSNs. We surveyed literature that attempts to proffer a solution to energy consumption issues with SNs during the network data transmission phase. With this in mind, we introduced a taxonomy to classify these energy-efficient and energy-balanced routing protocols for WSNs. Additionally, we classified these routing protocols based on the solution types or algorithms, and the design input decision variables. Also, we presented noteworthy areas for possible research directions.

An extensive survey on clustering routing protocols for WSNs is presented in [12,13,14,15,38,39]. In [12], the authors emphasised the challenges and logic in developing a clustering algorithm for WSNs. Also, the authors discussed the problems that face the practical design of clustering routing techniques in WSN applications. The nine surveyed papers in [12] are subsequently classified based on the clustering routing objectives and design principles. The authors in [13] introduced a taxonomy to classify clustering routing protocol; pinpointing the design complexity, objectives and principles. The authors [13] discussed the strengths and weaknesses of these clustering routing algorithms whereby they presented a comparison among these clustering techniques using some measures such as cluster stability, location-awareness, convergence rate. In [38], the authors focused only on energy-efficient hierarchical cluster-based routing protocols for WSNs. They discussed and compared different energy-efficient hierarchical cluster-based routing protocols for WSNs. The paper in [14] provides a survey on energy-efficient clustering routing protocols for heterogeneous WSNs. The authors compared fifteen heterogeneous energy-efficient routing protocols based on some factors such as clustering method, clustering attributes, location-awareness, and heterogeneity level. The authors in [39] classified clustering routing protocols based on their objectives, and methods of cluster head selection, cluster formation, data aggregation and data communication. They provided a taxonomy to the protocols in each of these phases. The authors also discussed the strengths and weaknesses of the techniques used in the studied clustering routing protocols. Finally, the authors summarised the issues and solutions of the attributes and characteristics of clustering approaches. In [15], the author presented the advantages and applications of clustering techniques in WSNs. In this regard, the author introduced a taxonomy to classify clustering routing protocols for WSNs. The author compared these clustering routing protocols based on different measures such as scalability, energy efficiency, cluster stability, and load balancing. On the contrary, we focus more on energy-efficient and energy-balanced routing protocols for WSNs. Interestingly, clustering techniques are extensively used to optimize energy consumption in WSNs. Thus, we review the clustering routing protocols that focus on optimizing the energy consumption in WSNs, and classified these clustering algorithms based on their mode of communication towards the BS. Our survey includes both homogeneous and heterogeneous energy-efficient and energy-balanced clustering routing protocols for WSNs. Additionally, we grouped these clustering routing algorithms, based on the cluster size formulation, into unequal and equal clustering routing algorithms.

Survey on multipath routing protocols for WSNs is presented in [16,17,18,19]. The authors in [16] explain the notion and challenges of multipath routing protocols for WSNs. They presented a taxonomy to classify the surveyed multipath routing protocols and provided the strengths and weaknesses of these surveyed multipath routing protocols. The authors [16] also provided a comparison to summarise these multipath routing protocols based on their network applications. In [17], the authors provided a survey on multipath routing protocols for WSNs. Afterwards, the authors classified these multipath routing protocols into three categories (infrastructure-based, non-infrastructure-based, and coding-based) and compared these multipath routing protocols based on these categories. The authors finally explained the evaluation metric, objectives, and challenges in designing multipath routing protocols for WSNs. The authors in [18] investigated the advantages of different multipath routing protocols and classified these surveyed multipath routing protocols based on their features. The survey in [19] compares different multipath routing protocols for WMSNs based on their working operations and provided the advantages and disadvantages of these multipath routing protocols. In contrast to the survey in [16,17,18,19], this paper pays attention to multipath routing protocols designed mainly to optimize energy consumption in WSNs. We review the multipath routing protocols developed to optimize energy consumption in WSNs. Accordingly, we classified these multipath routing protocols based on the solution types or algorithms, and the design decision variables used in the algorithms.

We therefore summarise the related survey work on WSNs in Table 1. The table provides the major relevance and contribution of each related survey work and the year of the survey. These surveys provide good insight into the applications, challenges and implementations of WSNs. Although some of the survey papers focus on energy-efficient routing protocols for WSNs, we provide a systematic survey on both energy-efficient routing protocols and load-balanced energy routing protocols. Moreover, we emphasised the importance and challenges of developing a load-balanced energy routing protocol. We discussed the pros and cons of the reviewed energy-efficient routing protocols and load-balanced energy routing protocols for WSNs. Subsequently, we classified these routing algorithms based on the solution types or algorithms, and design decision variables used in the algorithms. We envisage that this survey will help researchers and practitioners to make a decision on the right routing protocol in order to optimize the energy usage based on their application requirements and network formulations. Additionally, the paper can provide direction for new researchers in this field.

## 3. Terminologies Used in WSNs

In this section, we explain the terms generally used in the development of a WSN. Most importantly, some of these terms are used to classify the surveyed energy-efficient and energy-balanced routing protocols in subsequent sections. We expect that these explanations will give readers a better understanding of this survey paper and WSNs in general.

### 3.1. Network Lifetime

The main reason for the design of energy-efficient and energy-balanced routing protocols for WSNs is to extend the network lifetime, which in turn maintains the network functionality. The term "network lifetime" is a primary evaluation metric that is mostly used to measure the energy efficiency of a WSN. There are different definitions for the network lifetime of a WSN in the literature. Some literature defines the network lifetime as the time until the energy in any SN in the network is depleted or the time until the energy in all the SNs in the network is depleted. Whereas some authors refer to the network lifetime as the time until the energy in a defined percentage of the SNs in the network is depleted [40,41,42], other literature defines the network lifetime of a WSN based on the network application and formulation. However, the general idea remains the same. For the purpose of this survey, we simply define the network lifetime of WSNs as the maximum time that the network is capable of measuring a physical value or event.

### 3.2. SN Residual Energy

The lifetime of a SN is usually measured by its energy level after a given network data transmission round. The energy level in a SN after each network data transmission phase is referred to as its residual energy. Given that the energy of a SN at the initial deployment is defined as $E_{in}$, and the energy consumed by the SN after a particular data transmission round is $E_{r}$, the residual energy of the SN can be defined as:

(1) $$E_{re}=E_{in}-\sum_{r=0}^{R}E_{r},$$

where $E_{re}$ = $E_{in}$ for *r* = 0, *r* is the current round, and *R* is the maximum number of rounds. From Equation (1), when $E_{re}$ = 0*J*, the SN has completely depleted its energy and cannot participate in any network activity. Thus, the energy level of SNs is an important factor to be considered in order to maintain the functionality of the network for a reasonable time. In fact, as shown in Table 2, Table 3, Table 4, Table 5 and Table 6, the $E_{re}$ or (RE) is an important decision variable used in the design of most of the studied energy-efficient and energy-balanced routing protocols. The knowledge of the energy level of the SN becomes more important for the design of clustering-based routing protocols [43]. For example, the energy level of the CH node in C3 is zero ($E_{re}$ = 0*J*) as shown in Figure 2, therefore all the SNs in that cluster cannot participate in the next network activity. Hence, in order to avoid untimely network partitioning, most clustering-based routing protocols consider RE as an important decision variable in the routing algorithms.

### 3.3. Average Network Energy

The knowledge of average network energy is used by developers of WSNs to optimize the energy consumption by SNs. The average network energy at any given data transmission round can be expressed as:

(2) $$E_{av}=\frac{\sum_{1}^{n}E_{{re}_{n}}}{n},$$

where *n* is the total number of SNs in the network. The $E_{av}$ is widely used as a decision variable in designing energy-efficient and energy-balanced routing protocols for WSNs. In clustering-based routing protocols, the $E_{av}$ is defined as the energy threshold in selecting the CHs for a particular data transmission phase in order to avoid network segregation. Some other energy-efficient and energy-balanced routing protocols use $E_{av}$ or AE as the defined energy threshold in choosing the relay nodes towards the BS during that transmission. We studied and classified the different energy-efficient and energy-balanced routing protocol designs for WSNs that specify the AE as an input decision variable.

### 3.4. Distance Metric

The distance metric or hop-distance is an important parameter considered in the design of routing protocols for WSNs. The distance metric defines the distance between SNs or the distance between a SN and the BS. In clustering routing protocols, the distance metric defines the distance between a CH and a nCH, the distance between a CH and the BS, or in some cases, the distance between a nCH and the BS. Consider the clustering WSN of Figure 1; if the coordinates of $SN_{3}$ and $SN_{6}$ are defined as (A,C) and (B,D) respectively, the hop-distance between these nodes can be computed in the form of a distance metric as:

(3) $$d=\sqrt{{\left(SN_{3_{A}}-SN_{6_{B}}\right)}^{2}+{\left(SN_{3_{C}}-SN_{6_{D}}\right)}^{2}}.$$

In designing most routing algorithms, it is desirable that *d* is as small as possible in order to minimise the transmission energy. The distance metric *d* is directly proportional to the transmission energy. Hence, most energy-efficient and energy-balanced routing protocols strive to minimise the value of *d* in choosing the route(s) from the source node to the BS. The hop-distance is therefore an important decision variable in developing an energy-efficient and energy-balanced routing protocol.

### 3.5. Hop Count

The hop count (HC) is widely use as a decision variable in the design of energy-efficient and energy-balanced routing protocols for WSNs. In fact, some studies [44,45] used the hop count as an evaluation metric for comparing the performance of WSN routing protocols. The hop count is defined as the number of relay nodes traversed by the message from the source node to the destination. It is desirable to minimise the hop count during the data transmission phase in order to extend the network lifetime of WSNs as much as possible, but there is a compromise between reducing the hop count and maximizing the network lifetime of WSNs. By reducing the hop counts from the source node to the destination, some nodes are compelled to convey a large amount of load and can easily deplete their energy. Therefore, the hop count is broadly considered as a decision variable while designing a load-balanced energy routing protocol for WSNs.

### 3.6. Homogeneous and Heterogeneous WSNs

WSNs can be classified based on their infrastructure as homogeneous WSNs or heterogeneous WSNs. In homogeneous WSNs, all the SNs have similar hardware components such as the sensing subsystems, processing subsystem, radio subsystem, and power supply unit. On the other hand, the hardware components of two or more SNs are different in heterogeneous WSNs. A major reason for the design of heterogeneous WSNs is to equip some SNs with bigger sensing range and more battery power to attain longer transmission [46]. Although deploying homogeneous WSNs can be quite easy in comparison to heterogeneous WSNs, heterogeneous WSNs are more useful in factual deployments because they are close to practical scenarios [14]. Different energy-efficient and energy-balanced routing protocols have been proposed over the years assuming homogeneous or heterogeneous WSN structures. In some cases, the proposed energy-efficient and energy-balanced routing protocols are verified to be suitable for both homogeneous or heterogeneous WSN scenarios. We studied these energy-efficient and energy-balanced routing protocols and subsequently grouped them based on their infrastructural design as shown in Table 2, Table 4, Table 5 and Table 6.

## 4. Overview of Energy Consumption in WSNs

This section explains the energy dissipation by SNs during the network data transmission phase. We explain how SNs consume or conserve energy using a theoretical WSN energy dissipation model. Moreover, we illustrated how these SNs consume energy during the network transmission phase using an example of a typical WSN scenario. With this example, we explain the concept, objectives, and challenges of designing an energy-efficient and energy-balanced routing protocol which forms the basis of our classification.

### 4.1. WSN Energy Dissipation Model

**Figure 3:**
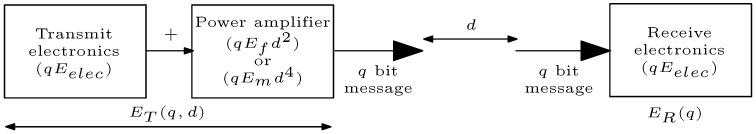
Simplified WSN energy dissipation model.

Consider the simplified WSN energy dissipation model illustrated in Figure 3 [47]. As shown in the figure, the transmitting SN consumes energy to drive its radio subsystem which includes the radio electronics, and power amplifier. The receiving SN also dissipates energy to drive its radio electronics. The distance *d* between the transmitting SN and receiving SN usually computed in the form of a distance metric specifies the channel model used by the power amplifier. If the distance *d* is greater than a set threshold $d_{0}$, the multipath model is assumed. Otherwise, the free space channel model is used for *d* < $d_{0}$. Therefore, the required energy for a SN to transmit a *q* bit message is defined as [48]:

(4) $$E_{T}\left(q,d\right)=\left\{\begin{array}{c} qE_{elec}+qE_{f}d^{2},\;d<d_{0}\\ qE_{elec}+qE_{m}d^{4},\;d\geq d_{0}, \end{array}\right.$$

where $E_{f}$ and $E_{m}$ are the free space and multipath power loss respectively, $E_{elec}$ is the energy dissipated to drive the radio electronics, and $d_{0}$ is the transmission distance threshold expressed as:

(5) $$d_{0}=\sqrt{\frac{E_{f}}{E_{m}}}.$$

On the other hand, the energy consumed by the receiving SN is defined as:

(6) $$E_{R}\left(q\right)=qE_{elec}.$$

These equations defined in (4)–(6) are used by developers of energy-efficient and energy-balanced routing protocols to theoretically verify their proposed algorithm because of their simplicity. However, in practice, we emphasise that the radio wave propagation varies rapidly and can be difficult to model using Equations (4)–(6).

### 4.2. SNs Energy Dissipation Issues

**Figure 4:**
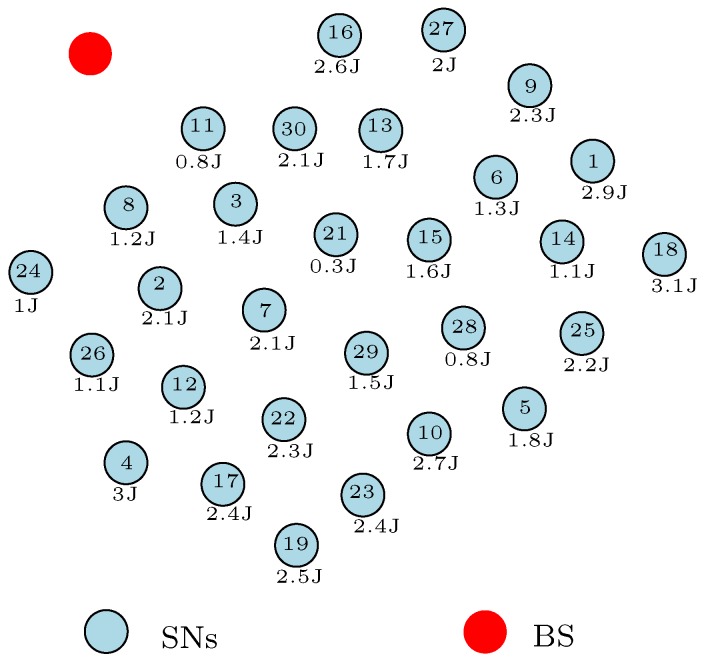
Typical WSN scenario, where *r* = 0.

Figure 4 depicts a typical WSN scenario where all the SNs are heterogeneous and static after their deployment. We also assume that the coordinates of the SNs and BS are known, and the residual energy of the SNs is as defined in the figure. From the figure, assume that $SN_{5}$ attempts to transmit a *q* bit message to the BS at its allocated time-division multiple-access (TDMA) schedule. Note that the TDMA schedule [49] makes certain that there are no data collisions during the network activities. In such a way, the TDMA schedule conserves energy by allowing SNs to sleep all the time except during the node’s transmission time. The $SN_{5}$ can convey the *q* bit message to the BS either by single-hop or multi-hop communication. In single-hop communication, $SN_{5}$ transmits the *q* bit message to the BS directly without using any relay node. As shown in the figure, we assume that the distance *d* between $SN_{5}$ and the BS computed in the form of a distance metric is considerably large. Therefore, applying Equation (4) means that $SN_{5}$ will consume a lot of energy to successfully transfer the *q* bit message to the BS. This forms a major disadvantage with single-hop communication. However, this can be advantageous in scenarios where the SN is very close to the BS. For example, this mode of communication will be advantageous if $SN_{8}$ attempts to convey its *q* bit message to the BS. Nevertheless, a major characteristic of WSNs is that the participating SNs are randomly placed in the sensor field, which means that some SNs will be at a huge distance from the BS. The SNs that are at a large distance from the BS will easily deplete their battery energy using single-hop communication to convey their messages which subsequently hampers the network functionality.

In an attempt to reduce the energy consumed by the SNs that are at a large distance from the BS, some energy-balanced routing protocols such as the LEACH (low-energy adaptive clustering hierarchy) protocol [50] clustered the WSN and rotated the duty of the CH. This method is somewhat useful because the distant nCH nodes do not need to send their sensed information to the BS directly. The nCH nodes send their sensed information to their respective CH with less transmission power in most cases as compared to sending the sensed information to the BS directly. This clustering technique, however, does not completely solve the distance problem associated with single-hop communication because the CHs convey the message from their cluster members directly to the BS. The placement of these SNs which include the CH is very unpredictable, and numerous works are ongoing to proffer a solution to the node placement problem with WSNs [51,52]. Therefore, in a scenario where the CH(s) is at a large distance from the BS, the CH nodes(s) will dissipate a lot of energy to transfer the message from its cluster members to the BS. These CH(s) can easily deplete their energy completely, thereby leading to the untimely partitioning of the network. As a result of this distance problem associated with single-hop communication, recent research work on WSNs makes use of the multi-hop communication method.

With multi-hop communication, the source SN transmits the *q* bit message to the BS through one or more relay nodes. In such a way, researchers envisaged that the energy dissipated by the SNs with large distances from the BS will be minimised in comparison to the single-hop communication method. The multi-hop communication method is used in different energy-efficient and energy-balanced routing protocols in order to optimize the energy consumption by the SNs in the network. Most of the energy-efficient and energy-balanced routing protocols in literature that are based on multi-hop communication can be categorized under (1) the clustering technique and (2) load-balanced tree technique. In the multi-hop clustering technique, the SNs transmit their sensed information to their respective CHs using multi-hop communication. Similarly, the CH sends the aggregated data to the BS via relay nodes (the relay node can either be a CH or nCH) or directly (single-hop communication) depending on the CH distance to the BS. The load-balanced tree technique finds the route from the source SN to the BS that balances the energy consumption in the network. The load-balanced tree technique can be in the form of a multipath algorithm or a single-path algorithm.

**Figure 5:**
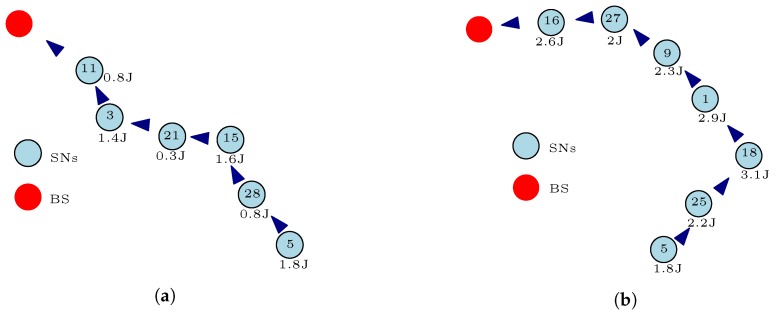
Multi-hop communication method. (**a**) Shortest path from *SN*_5_ to the BS; (**b**) Energy-efficient route from *SN*_5_ to the BS.

Nonetheless, these multi-hop communication methods have not completely solved the energy consumption issues with WSNs. A major challenge with these multi-hop communication methods is the trade-off between finding the most distance efficient path to the BS and the energy-efficient and/or energy-balanced path to the BS. For example, as illustrated in Figure 5a, we assume that the shortest path from $SN_{5}$ to the BS in Figure 4 is through $SN_{28}$, $SN_{15}$, $SN_{21}$, $SN_{3}$, and $SN_{11}$. As depicted, $SN_{21}$ has depleted more of its energy in comparison to the other SNs in the network. This simply means that $SN_{21}$ will die easily if used as part of the relay node when transmitting the message from $SN_{5}$ to the BS. In such a scenario, we can say that this distance efficient route to the BS from $SN_{5}$ is not the most “energy-balanced” route to the BS from $SN_{5}$. Although, as shown in Equations (4)–(6), the distance between SNs or a SN and the BS plays a key role in minimizing the energy consumption by the SNs; in this example, the distance efficient route does not offer a balance in the energy dissipated by the SNs in the network. Thus, finding the most distance efficient route to BS that optimizes the energy consumption by SNs can be a NP-hard problem because of network requirements and formulations.

Moreover, some energy-efficient routing protocols use the current energy level (residual energy) of the SNs in the network to find the most energy-efficient route(s) to the BS from the source node. The residual energy of SNs is a key design decision variable used by researchers to develop routing protocols for WSNs. Some of these algorithms select the SNs with high residual energy to form the relay node’s chain from the source node to the BS. For example, let us assume that the energy-efficient route from $SN_{5}$ to the BS in Figure 4 based on the energy level of the SNs is as represented in Figure 5b. These SNs acting as the relay nodes have high residual energy and can easily convey the message from $SN_{5}$ to the BS for many data transmission rounds before they completely deplete their energy. This energy-efficient route depicted in Figure 5b will definitely solve the problem associated with the distance efficient route of Figure 5a because the participating nodes can go many data transmission rounds before they deplete their energy completely. However, using these SNs with very high residual energy does not necessarily balance the energy consumption in the network. In this example, we assume that the sensed information from $SN_{5}$ is transmitted over a longer distance as compared to the illustration in Figure 5a. This means that the total energy consumed using this energy-efficient route depicted in Figure 5b will be more than the total energy dissipated using the shortest path example of Figure 5a with respect to Equations (4)–(6). In such a scenario, we can say that this energy-efficient route to the BS from $SN_{5}$ is not the most "energy-balanced" route despite the message being transmitted by relay nodes with high residual energy. Therefore, there must be a route from $SN_{5}$ to the BS that balances the energy dissipated by the SNs and the total energy dissipated in the network. This route can be very unforeseeable, and might change with each data transmission round depending on the energy level of the SNs in the network and the distance travelled in conveying the data.

Furthermore, an important observation in Figure 5a is that the energy-efficient route uses more relay nodes (hop count = 6) in conveying the message in comparison with the shortest path route (hop count = 5) in Figure 5a. As shown in Equation (6), this implies that the route in Figure 5b will dissipate energy more times (six times) to receive and convey the sensed message in comparison with the route in Figure 5a (five times). The hop count is a very important decision variable used by the developers of routing protocols for WSNs to optimize the energy consumption by the SNs in the network. Determining the optimal hop count when sending a message from the source node to the BS can also be a challenging problem with the multi-hop communication method. The smaller the hop count, the less energy consumed in receiving and relaying the message from the source node. As such, it is desirable to keep the hop count as small as possible between a source node and the BS, but this does not necessarily guarantee a balance in the energy consumption by SNs in the network. For instance, let us assume that the route in Figure 5a offers the smallest hop count from $SN_{5}$ to the BS. As shown in this figure, $SN_{21}$ forms part of the relay node’s chain in transmitting the message from $SN_{5}$ to the BS. However, the energy level of $SN_{21}$ is very low in comparison with the other SNs in the network as shown in Figure 4. At this energy level, $SN_{21}$ should not act as a relay node to $SN_{5}$ or any other node in the network in order to maintain the full network functionality. In fact, $SN_{21}$ should sleep all the time except when it is transmitting its own sensed information. Despite the fact that the route in Figure 5a offers the smallest hop count from $SN_{5}$ to the BS, we can say that this lowest hop count route is not the most “energy-balanced” route to the BS from $SN_{5}$. Thus, there must be a route with an optimal hop count from $SN_{5}$ to the BS that balances the energy consumed by the SNs and the total energy dissipated in the network.

Another obvious setback with the multi-hop communication method is the energy-hole problem [53]. In multi-hop communication, SNs closer to the BS consume more energy in receiving and forwarding the *q* bit message towards the BS. Because these SNs are randomly placed closer to the BS, most load traffic towards the BS passes through these nodes. As such, these nodes closer to the BS tend to dissipate more energy in comparison to the distant nodes. For illustration, most of the load traffic toward the BS in Figure 4 will pass through any of these nodes: $SN_{8}$, $SN_{11}$, $SN_{16}$, and $SN_{24}$. This implies that $SN_{8}$, $SN_{11}$, $SN_{16}$, and $SN_{24}$ have a high chance of completely depleting their battery energy in comparison to the other nodes in the network. Now, looking at the WSN in Figure 4 as a whole, the closer the nodes are to the BS, the more load traffic they carry, and as a result, they will consume more energy. This means that there is an imbalance in the energy consumption by SNs in the network. This imbalanced energy consumption will result in the untimely death of some SNs. The multi-hop communication routing protocol should be designed in such a way to mitigate the energy-hole phenomenon. Therefore, an energy-balanced multi-hop route from the source node to the BS must, at all times, solve the energy-hole problem related to the multi-hop communication method.

As mentioned, the design of a reliable energy-balanced routing protocol for WSNs involves a couple of decision variables (hop count, residual energy, distance metric, etc.) which must not be overlooked. The development of a routing protocol for WSNs that balances the energy consumption by the SNs in the network can be tricky because of the trade-offs in selecting these decision variables. However, the advantages of an energy-balanced routing protocol for WSNs cannot be overemphasised. This is more paramount in order to extend the network lifetime for a reasonable duration while also maintaining the full network functionality. The developers of energy-balanced routing protocols also consider other design objectives or advantages such as scalability, reliability, accuracy, and many more. Therefore, energy-balanced routing protocols for WSNs are sometimes developed to give a balance in some of these multiple desirable objectives (multi-objective) [54]. Yet, our attention is focused on the routing protocols whose main objective is to proffer a solution to energy consumption issues relating to WSNs. We analyse these energy-efficient and energy-balanced routing protocols based on the simple concept of how the SNs in the network dissipate energy during the data transmission phase. The technique(s) defined by the routing algorithm to conserve the energy dissipated by SNs when they convey their sensed information to the BS forms the basis of our classifications. Accordingly, we categorised the studied energy-efficient and energy-balanced routing protocol based on their mode of communication towards the BS. With this in mind, we also grouped these studied routing protocols based on the solution types or algorithms, and the input decision variables used to select the route to the BS from the source node.

## 5. Taxonomy for Energy-Efficient and Energy-Balanced Routing Protocols

Finding the energy-balanced route to the BS from the source node in order to maintain the network lifetime and functionality for a reasonable duration is the objective of all energy-efficient and energy-balanced routing protocols for WSNs. The techniques and approaches might differ, but the ultimate goal is to balance the energy consumption in the network in order to extend the network lifetime and functionality. As explained, different measures have to be considered in order to achieve this goal. Different research works on routing protocols for WSNs are ongoing to proffer a solution to its limited energy problem. We study and classify some of the state-of-the-art energy-efficient and energy-balanced routing protocols found in literature. The concept, objectives, and challenges of designing an energy-efficient and energy-balanced routing protocol as explained in Section 4.2 form the basis of our classification.

**Figure 6:**
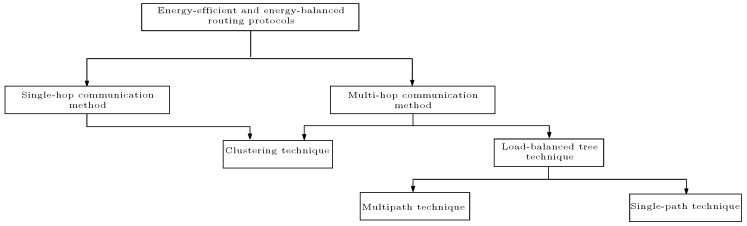
Classification of energy-efficient and energy-balanced routing protocols for WSNs.

The studied energy-efficient and energy-balanced routing protocols for WSNs are classified based on the their mode of communication towards the BS into the single-hop communication method and multi-hop communication method. As shown in the subsequent subsections, we realise and emphasise that all these studied energy-efficient and energy-balanced routing protocols for WSNs fall under any of these modes of communication as pictured in Figure 6. We explain the categories included in Figure 6, while also classifying the studied routing protocols under these categories. The studied energy-efficient and energy-balanced routing protocols are also grouped based on the solution types or algorithms, and design decision variables used in the algorithms. In addition, we discuss some of the advantages and disadvantages of these studied energy-efficient and energy-balanced routing protocols with respect to the choice of the input decision variables.

### 5.1. Multi-Hop Communication Method

A typical example of the concept of the multi-hop communication method is as shown in Figure 5. In multi-hop communication, the sensed information is conveyed to the BS from the source node using one or more intermediate nodes depending on the distance between the BS and the source node. Multi-hop communication has many benefits when used in WSNs. Besides improving the connectivity of a WSN and extending the network’s coverage, multi-hop communication helps in prolonging the network lifetime and functionality. In such a way, multi-hop communication allows higher transmission data rates and the effective use of the wireless communication channel. Different routing protocols or algorithms have been developed using the multi-hop communication method in order to optimize several single and multi-objective problems associated with WSNs. With heed to prolonging the network lifetime and functionality, researchers and practitioners have extensively used the concept of multi-hop communication to develop different energy-efficient and energy-balanced routing protocols for WSNs. The key reason for using the multi-hop communication concept is to reduce the distance in transmitting the sensed information from the source node to the destination. As mentioned earlier, the transmission distance plays a vital role in either reducing or increasing the energy dissipated by the SNs in the network. Nonetheless, solving the distance issue using the concept of multi-hop communication does not necessarily guarantee an optimal use of the limited battery energy in the network as explained in Section 4.2.

In an attempt to use the concept of multi-hop communication to solve the distance problem associated with WSNs, the multi-hop communication technique gave rise to other design issues such as selecting the route with optical hop count to BS and/or the route to the BS with optimal energy consumption (both in terms of the energy consumed by the participating nodes and the total energy dissipated using that route). Therefore, researchers came up with different multi-hop techniques to solve these problems, where the ultimate goal is to conserve the energy in the network. We classify these multi-hop techniques into two main categories: multi-hop clustering technique and load-balanced tree technique. In fact, all the state-of-the-art energy efficient and energy-balanced routing protocols studied fall under any of these multi-hop communication classes, if they are not based on single-hop communication. The concept and principle of these classes of the multi-hop communication method are discussed one after the other. Subsequently, we grouped the surveyed energy efficient and energy-balanced routing protocols accordingly, pinpointing their strengths and weaknesses. Finally, we grouped the surveyed routing protocols based on the solution types or algorithms, and the decision variables used in designing each of these algorithms.

#### 5.1.1. Multi-Hop Clustering Technique

**Figure 7:**
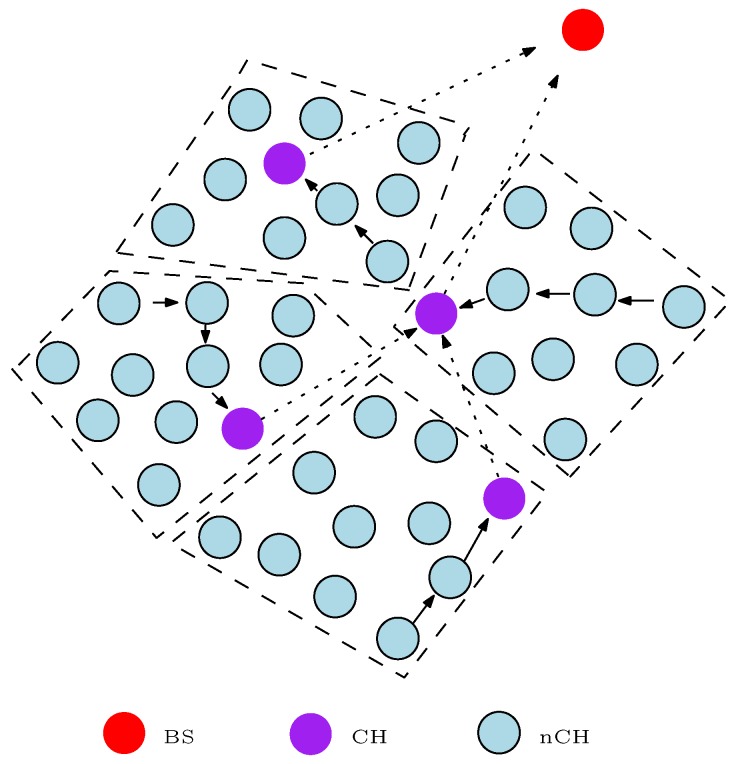
Typical example of a multi-hop clustering WSN.

Organising SNs into clusters has been broadly used by researchers to achieve different single and multi-objective optimization problems relating to WSNs. The clustering technique is widely used in designing routing protocols for WSNs because it has many advantages such as scalability, efficient data aggregation, fault-tolerance, latency reduction, robustness, and reduced energy consumption. A clustered WSN mostly contains two sets of nodes: the coordinating nodes usually referred to as the CH, and the member nodes known as the nCH, as shown in Figure 7. The CHs in the network are used to process the information before sending it to the BS while the nCHs forward the sensed information to their respective CHs. The clustering routing solution can be seen as a two-layer hierarchy where the CH nodes operate in the upper layer while the nCH nodes operate in the lower layer. As such, clustering routing protocols are usually referred to in the literature as hierarchical routing protocols. In some cases, because the CH nodes perform more functions than the nCH nodes, the CH nodes are equipped with a better sensing subsystem, processing subsystem, radio subsystem, and power supply unit in comparison with the nCH nodes. If the components of the CH nodes are different from the nCH nodes, the clustering WSN is referred to as a heterogeneous clustering WSN. Otherwise, researchers refer to the clustering WSN as a homogeneous clustering WSN. A clustering WSN can be based on either the single-hop communication method (refer to Section 5.2) or multi-hop communication method. In this paper, we refer to a multi-hop clustering WSN as a network whereby the message from the cluster members to their respective CHs and/or the message from the CHs to the BS is transferred using one or more intermediate nodes depending on the travel distance *d*. Therefore, we grouped the clustering energy-efficient and energy-balanced routing protocols for WSNs that require any form of intermediate nodes to convey the information either to the CHs or BS under the multi-hop clustering technique.

Multi-hop clustering techniques have been extensively shown to prolong the network lifetime, which is a primary evaluation metric for WSNs. The approach or design decision variables used by different researchers might differ but the clustering concept is unchanged. Basically, in the multi-hop clustering technique, the WSN is clustered as shown in Figure 7. The number of clusters or the size of the cluster is specified in the routing algorithm. The nCH nodes send the sensed information to their respective CH directly or via one or more relay nodes within their cluster. This clustering mode of communication is regarded as intra-cluster communication. The CHs aggregate all the sensed information from their respective cluster members and forward the aggregated information to the BS, either directly or via one or more relay nodes. In the case of the CHs, the relay nodes can either be a nCH node or a CH node. This means that any of the SNs in the network can serve as a relay node to convey the aggregated message from the CHs to the BS as specified in the routing algorithm. The communication between a CH and other clusters in order to convey the message from its cluster members to the BS is referred to as inter-cluster communication. Moreover, most clustering algorithms divide the network operation into rounds and periodically re-cluster the network in order to rotate the CH duty. The CH nodes consume more energy because they perform more functions in comparison to the nCH nodes. Hence, in order to avoid untimely network partitioning, it is always desirable to rotate the CH duty and select nodes with higher residual energy to operate as the CH in each round. By so doing, the load in the network is uniformly distributed among the SNs. Also, because the source node does not have to convey the *q* bit message over a long distance to the destination, the multi-hop clustering technique technically solves the distance problem affiliated with WSN, thereby conserving the energy in the SNs and the total energy in the network.

Despite the numerous advantages offered by the multi-hop clustering routing protocols [12,13,14,15], designing an energy-efficient and energy-balanced multi-hop clustering routing protocol for WSNs can be a challenging problem. A reliable energy-balanced clustering routing protocol carries some important attributes. Besides the general problems in designing multi-hop routing protocols briefly explained in Section 4.2, the design of a reliable energy-balanced multi-hop clustering routing protocol has its own peculiar energy dissipation issues, for example, assuming we decide to use a multi-hop clustering routing protocol for the WSN of Figure 4. The first major load balancing issue is to select the set of nodes that will perform the CH duty. As simple as it may sound, the CH selection phase strongly indicates the network lifetime performance of the proposed clustering routing protocol. As depicted and explained in Figure 2, if the CH for a particular cluster is inactive or it has completely depleted its battery powered energy, all the SNs in that cluster or sub-network have been segregated from the whole network. The SNs in that cluster will not be able to access the BS for that particular data transmission round despite having sufficient battery energy left. Thus, it is desirable that a SN with sufficient battery energy is always selected as the CH in order to avoid network partitioning and to maintain the full functionality of the network. In this regard, developers of clustering routing protocols pay careful attention in selecting the CH. The CH nodes are selected based on some design decision metrics. Some authors select the CHs based on the residual energy of the SNs [55,56,57,58,59,60,61,62,63,64,65,66,67,68,69,70,71,72,73,74,75,76,77,78,79,80,81,82]. They choose the SNs with the highest residual energy as the CH nodes, mainly because the CH consumes more energy than the nCH nodes. This approach sounds reasonable but might not be as efficient. If the nodes selected to act as CH are at a distant position from the BS and the clustering routing algorithm assumes that the CH communicates directly with the BS, then the CH node will use up more energy in forwarding the aggregated information from its cluster members to the BS . In [83,84,85,86,87,88], the authors select the CHs based on their proximity to the BS which is computed in the form of a distance metric. It is assumed that since the transmission distance *d* plays an important role in the energy dissipation model, the SNs with the shortest distances to the BS will consume less energy when forwarding packets to the BS. These selected SNs serving as CHs might be low on battery energy and if they are used continuously as CHs, they will completely deplete their energy, thereby resulting in untimely network partitioning. The authors in [89] selected the CHs based on their hop counts to the BS. As explained earlier, a small hop count from the source node to the BS helps to minimise the energy consumption in the network. However, this approach might suffer the same fate as using the shortest distance to the BS approach. Some authors select the CHs randomly based on some set threshold [90,91,92,93]. They set a threshold such that any SN that does not meet this condition cannot be a CH. The majority of these threshold-based CH selection methods define this threshold based on the estimated average energy or residual energy in the network. Thus, they randomly choose the CH nodes from the SNs that meet the set conditions. Moreover, some literature [94] firstly divides the network into a virtual grid. Subsequently, they select the gateways or CHs based on their location in the grid. They select the nodes based on their proximity to the boundary of the other grids in the network. This approach tends to prolong the network lifetime because more than one node can be used as gateways. As a result, the gateway function is shared. A setback with this approach is that the nodes acting as gateways are not rotated and might deplete their energy completely at some point during data transmission. As such, the grid(s) will be segregated from the whole network. We classified the literature that assumes that the location of the SNs is known in order to select the CHs under location-awareness (LA). Furthermore, some of the works in the literature select the CHs based on some mathematical formulations derived using a combination of some of these decision variables, thereby finding a balance in the trade-offs by using any of these metrics. In view of the aforementioned, Table 2 classifies the studied clustering routing protocols based on the common decision variables used in selecting the CH nodes. In the table, RE stands for residual energy, DBS stands for distance to the BS, HCBS stands for hop count to the BS, TB stands for threshold based, and TPBS stands for transmission power to the BS.

Note that the majority of these clustering routing protocols rotate the duty of the CH in order to balance the load in the network. Rotating the duty of the CH has been extensively shown to improve the network lifetime of clustering-based WSNs. Cluster head duty rotation strives to extend the network lifetime and functionality because the functions of the CH are shared among all the SNs in the network. Additionally, this approach avoids untimely network partitioning as shown in Figure 2. Table 2 showcases the reviewed routing protocol that offers this important load balancing feature in the multi-hop clustering technique. This CH rotation approach is more common with homogeneous clustering routing protocols as shown in Table 2. This is because all the SNs in the network assume the same battery energy at initial deployment. Rotating the CH duty in such a scenario will ensure that all the SNs share the heavy task of the CH, thereby evenly distributing the traffic load in the network. On the other hand, some of the heterogeneous clustering routing protocols assume large battery energy for the predefined CH nodes in comparison to the nCH nodes. Since these CH nodes have large battery energy, the CH duty is not rotated at any point. This approach tends to extend the network lifetime and functionality. However, the disadvantage is that, at some point, the CH nodes will completely deplete their battery energy because they perform more energy demanding functions. We classified the clustering routing protocols in which the CH is predefined and that are usually equipped with large battery power in comparison to the nCH nodes. As shown in Table 2, some authors avoid this disadvantage in heterogeneous clustering routing protocols by rotating the CH duty at some point during the data transmission phase. They define an energy level at which the CH duty is rotated among other SNs in the network. Moreover, some authors [95,96,97,98] propose a multi-sink technique to solve this problem. In this case, two or more sinks or BSs perform the CH duty. The sinks forward the aggregated information from their cluster members to the main BS. These sinks are powered in the same way as the main BS; thus, they do not have “energy consumption problems”. Although this approach solves the energy consumption issues with CH nodes, we assume it is not practical in reality. A major reason for our assumption is the practical cost implicated in deploying more than one BS to monitor an environment. This is not sustainable considering that WSNs are mostly designed to monitor a small geographical area. In this regard, we classified the studied energy-efficient and energy-balanced clustering routing protocols based on the assumed network infrastructure as a homogeneous (HM) and/or heterogeneous (HT) network. As shown in Table 2, we classified some algorithms that can be implemented in both heterogeneous and homogeneous network scenarios because the authors verified that their proposed clustering routing protocol is suitable for both scenarios.

Furthermore, in forming the cluster, an important aspect that must be carefully specified in the clustering algorithm is the approach in which the nCH nodes join a cluster. This aspect of the clustering routing algorithm is equally important when choosing the CH nodes. As marked in Table 2, most literature assumes that the nCH nodes join the cluster with the closest CH node to them in order to reduce the transmission distance. This approach technically reduces the energy consumed by the nCH nodes during data transmission because of the small transmission distance. However, since SNs are randomly placed in the sensor field, an obvious drawback with this approach is that some clusters might have excessive members in comparison with the other clusters. Therefore, the CHs carry more load traffic and will easily deplete all their battery energy. This results in an imbalance in the load distribution in the network. In lieu of this, some authors [60,99] strive to distribute the SNs to cover the whole sensor field by assuming that the SNs are aware of their location. This assumption might not be practical in reality because the approach is limited by location services in the network but it enables better sensing or measurement of the physical value as the SNs are evenly distributed around the sensor field [100]. As a result, different research works are ongoing to solve the node placement problems relating to WSNs [51,52]. Moreover, the hop count can be used as a decision variable to specify the cluster that a nCH node joins. As shown in Table 2, some literature uses the hop count as a yardstick for a nCH node to join a cluster. The nCH node compares the hop count to convey information to every CH in the network and joins the cluster with the smallest hop count. The hop-count approach reduces the energy consumed in receiving and relaying the information from the source node to the CH but will suffer the same setback as the shortest distance approach. Another decision variable used by researchers to specify the cluster that a nCH node joins is the received signal strength or transmission power. In this case, the nCH node estimates the transmission power required to convey a message to all the CH in the network; thus, it joins the cluster with the smallest transmission power. Similarly, this approach will suffer the same setback as the shortest distance approach. Note, from Equation (4), that the transmission power is a function of the distance between two nodes. This technically means that a nCH node will join a cluster with the closest CH node to it. In addition, in some algorithms, the nCH nodes join clusters based on a probability that depends on their residual energy. However, some literature specifies that the cluster that a nCH node joins is based on some mathematical formulations derived using a combination of one or more decision variable in order to effectively distribute both the inter-cluster and intra-cluster load traffic. In short, the assumptions, concepts, and techniques used by different authors to distribute SNs into clusters play a salient role in effectively balancing the load traffic in the network. As such, in Table 2, we grouped the surveyed energy-efficient and energy-balanced clustering routing protocols based on the common decision variables used to distribute the nCH nodes into clusters. In the table, DCH stands for distance to the CH, HCCH stands for hop count to the CH, TPCH stands for transmission power to the CH, while the clustering algorithms that do not allot the nCH nodes into clusters using any of the aforementioned decision variables are assigned under “Others”.

Having selected the CH nodes and specified how the nCH nodes should join a cluster, the next load-balanced requirements with the clustering routing protocol technique are (1) how to partition the network into an optimal number of sub-networks or clusters, and (2) the optimal cluster side. Partitioning the WSN into sub-networks is a noteworthy area of research. Some literature simply divides the network randomly into equal sizes while other works attempt to partition the network into different shapes [60,69,121]. Besides, some research work divides the network by assuming a virtual grid-based network structure [94,139]. Much more research is still ongoing in order to proffer a solution on how to efficiently divide the WSN into clusters, whereby the ultimate goal is to prolong the network lifetime. In addition, much literature has focused on determining the number of clusters and the optimal cluster size [75,86,101,108]. These papers strive to distribute the the SNs into clusters so as to have an equal cluster size. A general assumption in some of this literature is that an equal cluster size helps in balancing the traffic load in the network, most especially the intra-cluster traffic load. This might actually be the case but some literature argues otherwise. In [59,60,61,62,105,115,131,138], the authors explained how an equal cluster size results in an imbalance in the energy consumption by the SNs in the network. Basically, it is perceived that equally distributing the SNs to clusters is a good approach to balance the intra-cluster traffic load; however, this approach suffers during inter-cluster communications. The clusters closer to the BS consume more energy in receiving and forwarding the load traffic from other distant clusters during inter-cluster communications. This results in an imbalance in the energy consumption during inter-cluster communications and can result in early partitioning of the network. This phenomenon is also termed the energy-hole problem [53].

An efficient approach used by researchers to mitigate the energy-hole problem while developing a clustering routing protocol is to divide the network into unequal clusters. The size of each cluster depends on how close the cluster is to the BS. That is, the cluster size increases as the distance of the CH nodes from the BS increases. With this in mind, we classified the studied energy-efficient and energy-balanced clustering routing protocol, based on the clustering formation, into equal or unequal cluster size as shown in Table 3. Besides, some literature strives to mitigate the energy-hole problem by assuming, in the clustering routing protocol, that the BS or sink is mobile. In [139,140,141], the authors assume that the BS moves across the sensor field in order to change the direction of the load traffic. In so doing, it is assumed that all the SNs in the network randomly participate in forwarding the load traffic to the BS; thus, the energy consumption is distributed equally among all the SNs. Chiefly, with the mobile BS approach, the BS moves back and forth around the sensor field so as to collect the aggregated information conveyed from each CH. The mobile sink approach is still a very recent technique used in solving the energy-hole problem but has been shown to protract the network lifetime and functionality [139,140,141]. However, a major setback to this approach is that the BS has to continuously broadcast its current position to the SNs in the network. This results in high network delay due to the complexity in sending the present position of the BS.

Although the cluster formation phases are very important in order to balance the load traffic and subsequently maintain the WSN functionality for a reasonable period, the main focus of this paper is to study how the network load is balanced during the data transmission phase. Having discussed how the aforementioned multi-hop clustering routing protocols carry out the clustering formation steps, we study the decision variables used by these clustering routing protocols in setting up the multi-hop route from the source node to the BS. As explained earlier in Section 4.2, the proposed approaches used in selecting the route from the source node to the BS play a vital role in balancing the traffic load in the network. This, in turn, conserves the energy of the SNs in the network and the total energy in the network. We divided our classification of these multi-hop clustering routing protocols into two phases. We grouped the clustering routing protocols based on the decision variables used during the intra-cluster communications and the inter-cluster communications as shown in Table 3. From the table, some literature defines the intra-cluster communication routes or relay node’s chain towards the CH based on the distance usually computed using Equation (3). Researchers strive to reduce the transmission distance by selecting the nearest node to the CH node. This approach tends to conserve the energy dissipated by the forwarding nodes as a result of the small transmitting distance. However, this approach does not necessarily balance the load in the network. Using the illustration of Figure 5a, the shortest path or distance route to the CH is not always the most energy-balanced route. Some of these relay nodes along that route might be low on battery energy. Subsequently, such nodes should be protected as much as possible from performing relaying duties, or else, the nodes will deplete their battery energy completely. Moreover, for example, given three nodes X, Y, and Z—assuming the nearest node to X towards the CH is Y, and that node Z is next to node Y—node X will send the sensed information to node Y; node Y will subsequently forward the message to node Z, and so on. Nonetheless, it is possible that node X will conserve the total energy dissipated in the network by transmitting the information directly to node Z. That is, the energy dissipated in transmitting the information from node X→Y and Y→Z is greater than the energy dissipated in transmitting the information directly (without using node Y) from X→Z. As such, the shortest distance route might not always be the most energy-balanced route to the CH. Besides, as shown in Table 3, some papers define the intra-cluster communication routes based on the hop count or nearest hop to the CH. Similarly, this method conserves the energy in the network—most especially the energy dissipated by the source node—but this method has the same weaknesses as the shortest distance method. In the table, some authors select the most energy-efficient route to the CH. In the algorithms, the authors use the residual energy of the SNs as a key design decision variable in selecting the routes. The algorithms select the SNs with high residual energy to form the relay node’s chain from the source node to the CH. As emphasised with the illustration in Figure 5b, if the travelling distance in sending the information between the participating nodes toward the CH is unreasonably high, the total energy dissipated in the network will equally increase. In addition, some multi-hop clustering routing algorithms transmit the message from the source node to the CH directly as shown in Table 3. The authors only use the multi-hop technique during the inter-cluster communications. Note, in Table 3, all these clustering routing protocols use the multi-hop communication method during either intra-cluster or inter-cluster communications. In most cases, the multi-hop communication method is used in both the intra-cluster and inter-cluster communications. The clustering algorithms studied in this paper that do not use the multi-hop communication method are classified in Table 6 under single-hop communication. Also, some of the studied clustering algorithms select the relay nodes during the intra-cluster communications based on a probability that depends on two or more of the aforementioned design decision variables. The algorithms attempt to balance the trade-off in selecting the most energy-balanced route to the CH. For instance, as shown in Table 3, some literature jointly uses the transmitting distance between SNs and the residual energy of the SNs as the input decision variables in selecting the relaying nodes during the intra-cluster communications. In so doing, they reduce, to the nearest minimum, the disadvantages of using either the shortest distance or residual energy only in selecting the relay nodes. The disadvantage with some of these approaches is the high network delay as a result of the complexity in the proposed mathematical formulations. Furthermore, some of these studied clustering routing protocols concentrated more on the clustering formation. Since the focus of these papers is on producing a load-balanced cluster, the authors do not give a clear explanation on the intra-cluster and inter-cluster communications. The mode of communication used in transmitting the sensed information towards the BS in these papers can either be the multi-hop or single-hop communication method. However, we classified these papers under multi-hop communication as shown in Table 2 and Table 3. Additionally, as shown in Table 3, since the design decision variables used for both the intra-cluster and inter-cluster communications in these papers are not mentioned, we grouped the papers under “Cluster only”. Also, we classified the clustering routing protocols that do not use any of these mentioned design decision variables in Table 3 under “Others”. Lastly, we classified any other design decision variables not mentioned in Table 3 that are used in selecting the routes to the CH and/or BS, together with those mentioned in Table 3, under “Others”.

Likewise, we classified the design decision variables used in selecting the route to the BS during the inter-cluster communications. In a similar way to the intra-cluster communication classifications, we grouped the inter-cluster communications under “Direct”, “Residual Energy (RE)”, “Distance”, “Hop count”, and “Others”. Another important factor to be considered during the inter-cluster communication phase is the tier of the SNs acting as part of the relay node’s chain in forwarding the aggregated information from a CH node to the BS. Since clustering WSNs are regarded as a two-tier hierarchical network, the tier of the node used to form the relay node’s chain towards the BS plays a vital role in balancing the load traffic in the network. Some authors use the upper tier nodes (CH nodes) only to form the relay node’s chain towards the BS. Although this approach is better than sending the aggregated data directly over a large distance to the BS, the approach tends to increase the work load of the CH nodes. In this case, beside aggregating and forwarding the information from their respective cluster members, the CH nodes are given the sole responsibility of also serving as relay nodes in forwarding the aggregated information to the BS. Accordingly, this increases the duty of the CH nodes, thereby increasing the energy dissipated by the CH nodes in that particular data transmission round. Moreover, the CH nodes close to the BS will consume more energy because they always have to participate in the relaying duties. On the other hand, since the CH nodes perform more duties in comparison to the nCH nodes, the lower tier nodes (nCH nodes) can be used to form the relay node’s chain towards the BS. As shown in Table 3, this approach has not been used but we emphasised that this approach will reduce the burden of the CH nodes in relaying the message to the BS. Therefore, we assumed that this approach can be considered in designing multi-hop clustering routing protocols in the future. Moreover, some authors share the relaying duties between the two-tier nodes. Both the CH nodes and the nCH nodes can act as a relay node in transmitting the aggregated information towards the BS depending on the design decision variables used. This approach tends to prolong the network lifetime performance as compared to using only the CH nodes. However, if the design decision variables are not carefully specified in the clustering algorithm, the SNs acting as CH nodes might frequently participate in the relaying duties. Thus, this approach will suffer the same setback as using only the CH nodes. With regards to this, we grouped the studied clustering routing protocols that employ the multi-hop method during the inter-cluster communication phase based on the nodes performing the relaying duties toward the BS. As shown in Table 3, we grouped the relay nodes used during the inter-cluster communication phase under “CHtoBS”.

Finally, in Table 3, we grouped the studied multi-hop clustering routing protocols based on the solution types or algorithms used. Optimization plays a vital role in WSNs. Generally, an optimization problem comprises of the input decision variables, constraints, objective functions and the outputs. In most of the optimization problems associated with WSNs, these component parts can be merged with numerous dissimilar combinations resulting in different kinds of optimization problems. As such, there is no single algorithm that proffers a solution to the different optimization problems affiliated with WSNs [54]; this is with respect to the energy consumption issues with WSNs, where the major objective function is to minimise the energy consumption in the SNs in the network and the total energy in the network. We grouped the studied multi-hop clustering routing protocols based on optimization solution types or algorithms as shown in Table 3. As illustrated in Table 3, “B” stands for bio-inspired-based algorithms, “E” stands for evolutionary-based algorithms, “G” stands for game theory-based algorithms, “H” stands for heuristic-based algorithms, “L” stands for linear programming-based algorithms,“M” stands for meta-heuristic-based algorithms, and “S” stands for stochastic-based algorithms.

#### 5.1.2. Load-Balanced Tree Technique

**Figure 8:**
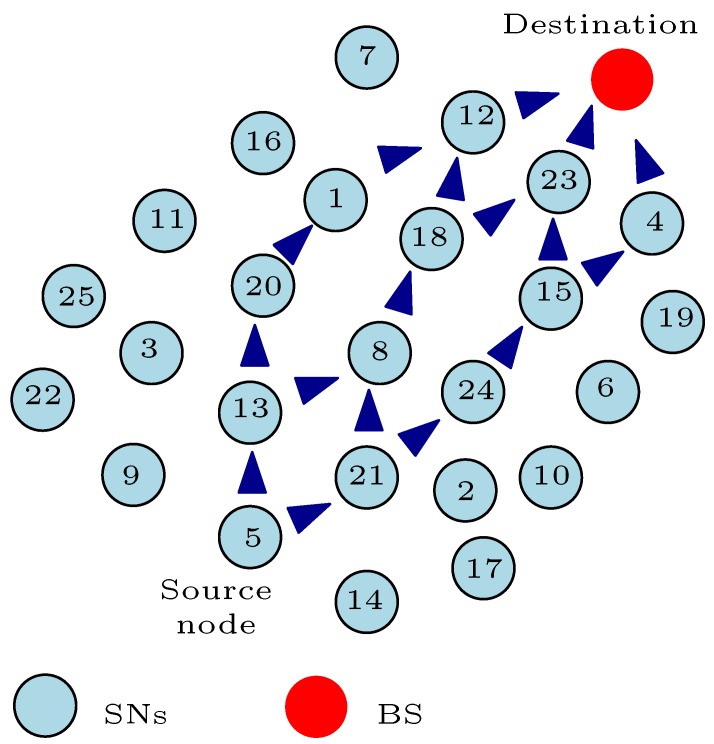
Typical example of multipath load-balanced tree routing.

**Figure 9:**
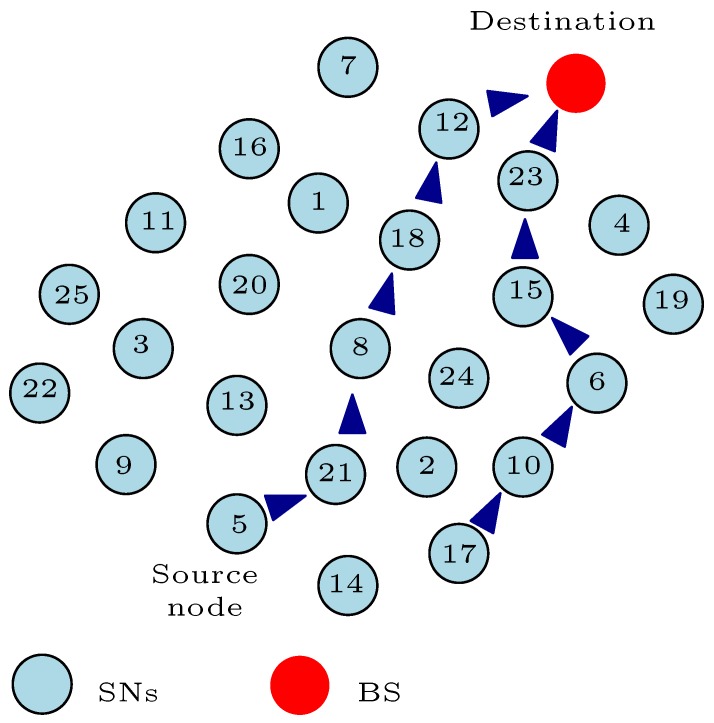
Typical example of single-path load-balanced tree routing.

The load-balanced tree technique finds an energy-balanced and energy-efficient path(s) from the source node to the BS. A typical illustration of the load-balanced tree technique is shown in Figure 8 and Figure 9. In Figure 9 for example, the source nodes ($SN_{5}$ and $SN_{17}$) construct an energy-efficient and energy-balanced route to the BS. The route from the source node to the BS resembles a tree structure. Literally, a tree is made up of three parts: the root, the trunk, and the crown. In WSN terms, the root represents the source nodes, the trunk can be regarded as the intermediate nodes, and the crown technically represents the BS. Thus, as shown in Figure 9, the sensed information (which can be seen as the nutrient from the soil) is forwarded through the trunk (intermediate nodes) to the crown (BS) of the tree. In order to extend the network lifetime and functionality, researchers strive to balance the load from the root to the crown of the tree. Accordingly, in a load-balanced tree routing protocol, much attention is on the design of the trunk (relay node chain to the BS). The route construction can be in form of a multipath technique (Section Multipath Technique) or a single-path technique, as discussed in Section Single-Path Technique. These constructed route(s) often change in each data transmission round. The concept is that the load traffic carried by the source node is always evenly distributed as much as possible in each data transmission round [142]. In such a way, the bulk of the load traffic will not flow through the source node only, and the direction of flow to the BS changes all the time depending on the routing algorithm. The load-balanced tree approach has been extensively used to design a WSN routing protocol because it offers numerous advantages as discussed in the subsequent sections. In lieu of this, we study and classify some of the load-balanced tree routing protocols in the literature.

##### Multipath Technique

Multipath routing is a load-balanced tree technique broadly used by researchers to develop routing protocols for WSNs in order to improve the performance of the network. The multipath routing approach has been used extensively in the past decade for different optimization problems in WSNs such as load balancing, fault-tolerance, bandwidth aggregation, security, data transmission reliability, and congestion control. Multipath routing is a routing approach that chooses multiple paths to convey data from the source node to the destination in the form of a tree as shown in Figure 8. As in the case of the multi-hop clustering technique, all multipath routing protocols fall under the multi-hop communication method. As shown in Figure 8, the source node uses different relay nodes to convey its sensed information to the sink. However, due to the dense placement of the SNs, several paths can be constructed from the source node to the destination. Multipath routing techniques are initially established to proffer solutions to the low data transmission rate associated with the single-path routing method. Nowadays, multipath routing techniques are used to proffer solutions to different WSN’s optimization problems as mentioned. With heed to load balancing, multipath routing achieves load balancing by distributing the load traffic from the source node over more SNs towards the destination. For example, consider the expressions in Equations (4) and (6), where the load traffic is defined by *q*. As shown in the equations, the load traffic *q* (message bit) is directly proportional to the energy consumption. That is, the more the load traffic *q*, the higher the energy consumed by SNs in transmitting or receiving the message. Thus, with multipath routing, the traffic load is shared among the SNs from the source node towards the destination. In such a way, the network lifetime and functionality are prolonged for a reasonable period.

In spite of the numerous advantages offered by multipath routing protocols, designing a multipath routing protocol can be a challenging problem. A good multipath routing protocol takes into consideration several components to select the multiple paths and evenly distribute the traffic load over the discovered paths. The main components to be addressed in designing a multipath routing protocol include path discovery, path selection/load distribution, and path maintenance [16]. Since multipath routing is designed based on the multi-hop communication principle, the duty of the path discovery stage is to select the set of relay nodes that will form the multiple paths from the source node towards the destination. After the set of intermediate nodes has been selected, the number of paths (path selection) to distribute the traffic load is also another vital component. Furthermore, as a result of resource constraints and the low battery power of the SNs, the selected paths are usually susceptible to error. Therefore, path maintenance is an essential component in designing a multipath routing protocol. Path maintenance helps to ensure reliable data transmission from the source node to the destination which is an important advantage of multipath routing protocols.

Since the focus of this paper is on energy consumption reduction, we based our classification of the studied multipath routing protocols on the path discovery and path selection/load distribution steps as shown in Table 4. The importance of the path discovery and path selection/traffic distribution phases cannot be overemphasised in designing an energy-efficient and energy-balanced multipath routing protocol. During the path discovery phase, the set of relay nodes that will participate in constructing the multiple paths to the BS is selected. Researchers select the set of intermediate nodes based on different parameters. Some literature selects the participating relay nodes based on their residual energy (RE). This approach tends to prolong the lifetime of the SNs with low battery life. Nevertheless, this approach does not necessarily conserve the energy in the network during a particular data transmission round. This is because the participating nodes with a good energy level might be at large distances from each other. Thus, the SNs will consume more energy in relaying the sensed information to the BS. As such, some authors select the participating SNs based on other parameters such as hop count (HC), hop-distance, received signal strength/transmission power (RSS/TP), etc. All these mentioned design decision variables have their pros and cons when used in selecting the relaying nodes that will form multiple paths to the BS. For instance, using the distance between nodes as the yardstick in selecting the relaying nodes tends to reduce the transmission power; consequently, it minimise the energy consumption in conveying the sensed information. A drawback with this approach is that some of the participating nodes might be low on battery power and they will completely deplete their battery power when used consistently. Similarly, the HC will suffer the same fate as using the shortest distance approach when it is assumed as the design decision variable. In this regards, as shown in Table 4, most of the authors find a balance in using these decision variables. The authors select the set of intermediate nodes based on some mathematical formulations derived using a combination of some of these decision variables. Therefore, we grouped these multipath routing protocols based on the decision variables used in the path discovery phase as shown in Table 4. The multipath routing protocols that do not use any of these mentioned design decision variables in Table 4 are grouped under “Others”. Besides, we classified any other design decision variables not mentioned in Table 4 that are used in selecting the set of relay nodes, together with those mentioned in Table 4, under “Others”.

The path selection/load distribution phase is very important in designing an energy-efficient and energy-balanced multipath routing protocol. During this phase, the number of paths to distribute the traffic load is firstly determined. Subsequently, this phase aims to reduce the energy consumption in distributing the load from the source node towards the destination. Choosing an adequate number of paths is vital in the design of a reliable multipath routing protocol. A multipath routing protocol may use only the most cost efficient path for data transmission and the other paths for fault-tolerance [16,159,160,161]. On the other hand, some multipath routing protocols use multiple paths synchronously to provide an evenly distributed traffic load or a reliable data transmission [162,163,164,165]. Thus, the number of chosen paths is important in order to improve the performance of the attributes of multipath routing protocols. Once the given number of paths has been selected, the multipath routing protocol finds a way to evenly balance the network load over the chosen paths. In Table 4, we classified the studied multipath routing protocols based on the parameters that the authors employ in distributing the network load over the selected paths. As shown in the table, authors of the studied multipath routing protocols consider parameters such as RE, HC, total transmitted energy (TE), transmission distance, and data transmission rate in distributing the network over the chosen paths. These parameters must be carefully selected because they all offer different advantages and disadvantages. For instance, using the RE as the decision variable in selecting the paths will prolong the network lifetime for a reasonable duration while also ensuring a reliable data transmission. This is because more load is distributed to the paths where the participating SNs have a good energy level. As such, it is guaranteed that the sensed information will reach its destination since all the nodes along that path have sufficient energy to receive and transmit the sensed information towards the destination. A common disadvantage associated with this approach is the transmission distance problem. This high RE path might require a large distance to eventually convey the sensed information to the BS. As explained in Section 4.1, the larger the distance, the more energy consumed in conveying the *q* bit message. Some authors ignore the energy level of the SNs while distributing the load over the selected paths. They distribute the load based on the assigned rates on each path. In this case, they distribute more load to the paths with a high data transmission rate. This approach does not guarantee a load-balanced WSN or a reliable data transmission because the participating nodes might be low on battery energy and will deplete their battery energy completely during transmission. However, the approach avoids delay in data transmission which is very useful during real-time data transmission. Moreover, in order to reduce the transmission distance, thereby minimising the energy consumption, some literature uses decision variables such as the HC and the hop-distance to distribute the load traffic over the selected paths as shown in Table 4. Although the HC or hop-distance approach tends to reduce the energy consumption, even in real-time, both approaches suffer if the participating SNs have low residual energy. The authors in [143,145] consider the total transmitted energy by each path as part of the decision variables before distributing the network load to these selected paths. The authors firstly measure the energy consumed by each path to convey the sensed information to the BS based on the distances between the participating nodes in that path. Subsequently, they distribute more load to the paths that will consume less energy in conveying the message to the BS. This approach will prolong the network lifetime if used with other parameters such as the RE as in the case of [143,145]. For example, let us assume that this approach is used only in distributing the load over the selected paths. Accordingly, more load is distributed to the path that is measured to consume less energy in conveying the sensed information. If the participating nodes along that path are low on battery energy, they will deplete their energy completely during data transmission. As a result, the network functionality has been hampered. Therefore, as shown in Table 4—regarding the studied multipath routing protocols—most of the authors find a balance in selecting these decision variables while distributing the load over the selected paths. Furthermore, we grouped the multipath routing protocols that do not use any of these mentioned design decision variables in Table 4 under “Others” while any other design decision variables not mentioned in Table 4 that are used in distributing the load over the selected paths, together with those mentioned in Table 4, are classified under “Others”.

Additionally, in Table 4, we classified the studied multipath routing protocols based on the assumed network infrastructure as homogeneous and/or heterogeneous WSNs. Moreover, some of the studied routing protocols assume that the SNs are aware of their locations in selecting the multipath. Although, as explained earlier in Section 5.1.1, assuming that the SNs are aware of their locations may not be practical in reality; we classified the studied multipath routing protocols that base their design on this assumption. Finally, we classified the studied multipath routing protocols based on the optimization solution types or algorithms as shown in Table 4.

##### Single-Path Technique

The single-path technique is another class of the load-balanced tree routing protocol. In this case, the source node finds a single energy-efficient and energy-balanced route to the BS as depicted in Figure 9. The single-path technique is easy to implement and exhibits low complexity in comparison to other multi-hop communication methods. Moreover, the technique avoids delay in conveying the sensed information to the BS, which makes it very suitable in real-time communication. Other advantages of the single-path load-balanced tree technique include congestion and interference control, and minimum resource utilization. Although the design of a single-path load-balanced tree routing protocol can be quite simple in comparison to the other multi-hop communication methods, selecting the design decision variables used in the path creation can be a challenging problem, as in the case of the other multi-hop communication methods. Besides choosing an energy-efficient and energy-balanced path from the source node to the BS, the chosen path must guarantee reliable data transmission. Since the message is routed through a single path, the path must be reliable because there is no room for fault-tolerance, as in the case of the multipath technique. As such, the design decision variables must be carefully selected in order to ensure reliable data transmission and to extend the network lifetime for a rational period. Therefore, most of the studied single-path routing protocols select the path to the BS using different mathematical formulations. These mathematical formulations are derived using different combinations of the mentioned decision variables in Table 5. As represented in Table 5, the design decision variables used by these single-path multi-hop routing protocols to select an energy-efficient and energy-balanced path to the BS are grouped under “residual energy (RE)”, “hop-distance”, “hop count (HC)”, “total energy (TE)”, “received signal strength/transmission power (RSS/TP)”, “throughput” and “average network energy (AE)”. These parameters have different strengths and weaknesses; hence, researchers attempt to balance the trade-offs in their selection. For example, the authors in [166,167] balance the trade-off in using the residual energy and the distance between nodes in selecting the path from the source node to the BS. Their algorithms forward the sensed information to the nearest node with high residual energy. In so doing, they firstly reduce the transmission distance; the algorithms also ensure that the forwarding SN has sufficient energy to convey the sensed message, which equally guarantees a reliable data transmission. Having mentioned the advantages of the approach in [166,167], using the residual energy and shortest distance between nodes as criteria does not necessarily balance the load in the network; this approach might involve many hops or relay node chains to reach the BS. Thus, more energy will be consumed in receiving (Equation (6)) and conveying (Equation (4)) the sensed information to the BS. This implies that the total energy consumed in the network might increase. So, it is desirable to keep the hop count between the source node and the BS as small as possible. With this in mind, some literature [168,169,170] combines the RE, distance, and HC in selecting the path from the source node to the BS. In addition, as shown in Table 5, while some authors combine two or more decision variables in choosing an energy-efficient and energy-balanced route, some authors use a single decision variable to select the path from the source node to the BS. The authors in [171,172] use the hop count and distance respectively as the only decision variable in selecting the path between the source node and the BS. Likewise, the authors in [173] use the throughput only to select the next hop. Their algorithm [173] alternately selects the nodes with the highest throughput as the next hop towards the BS.

Although the chosen decision variables in these papers [171,172,173] have their advantages, important decision variables such as the RE are not taken into consideration in choosing the next hop. As mentioned earlier, the importance of the RE in the design of an energy-efficient and energy-balanced routing protocol cannot be overemphasised. If the next hop node in these algorithms [171,172,173] is low on battery energy, the SN will deplete its energy completely while receiving or conveying the sensed information. Additionally, some of the authors in Table 5 assume that the SNs are aware of their location in designing their routing algorithm. Consequently, we grouped the single-path routing protocols that assume location-awareness in their design. Besides, as illustrated in Table 5, we group the studied single-path load-balanced tree routing protocols based on the assumed network infrastructure as homogeneous and/or heterogeneous WSN. Lastly, we classify the studied single-path routing protocols based on the optimization solution types or algorithms as shown in Table 5.

### 5.2. Single-Hop Communication Method

**Figure 10:**
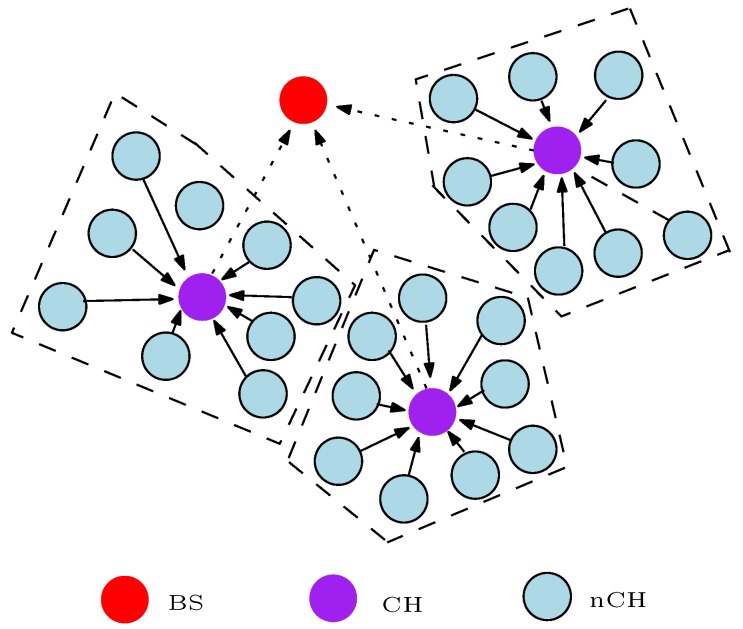
Typical example of a small-scale single-hop clustering WSN.

In the single-hop communication method, the sensed information is conveyed to the BS from the source node directly. This method was largely used in the early discovery phase of WSNs. Yet, the single-hop communication method offers some advantages. For instance, the single-hop communication approach avoids delay in data transmission which is a useful attribute in real-time data transmission. The single-hop communication method also avoids the energy-hole phenomenon which is an important advantage for using this method in designing a routing protocol. Besides, the single-hop communication method ensures reliable data transmission while all the SNs are still alive. In the multi-hop communication method, part of the sensed information might be lost while conveying the message from one node to another towards the BS. On the other hand, in the single-hop communication method, the message is sent directly to the BS. Conveying the message directly to the BS technically avoids the situation whereby part of the sensed information is lost in the process of sending the information from one node to another. Nevertheless, the single-hop communication method is not suitable in a large-scale WSN. In such a network, the distant SNs will always send their sensed information to the BS over a large distance. Thus, they will deplete their battery energy quickly in comparison to the nearby SNs. Accordingly, the network lifetime and functionality will be hampered. So, in an attempt to extend the network lifetime and functionality for a reasonable period, researchers proposed different single-hop clustering routing protocols, as shown in Table 6. These single-hop clustering routing protocols strive to optimize several single and multi-objective problems associated with WSNs. Figure 10 depicts a typical example of a single-hop clustering WSN. As shown in the figure, the nCH nodes send their sensed information to the CH directly without the help of any intermediate node. Similarly, the CH nodes forward the aggregated data from their cluster members directly to the BS, irrespective of their positions from the BS. This single-hop clustering approach is somewhat useful because all the SNs do not need to send their sensed information to the BS directly. The nCH nodes send the message to their respective CH nodes which, in most cases, are at a shorter distance from them in comparison to the BS. The single-hop clustering approach does not necessarily solve the distance problem associated with the single-hop communication method. For example, in a large-scale WSN, some nCH nodes might be at a large hop-distance from their respective CH nodes. More realistically, some of the CH nodes in such a network will be at a large distance from the BS. If these CH nodes continuously send the aggregated data to the BS directly, they can easily deplete their battery energy completely. This will result in early network partitioning as explained in Figure 2. However, we studied some single-hop clustering routing protocols based on the cluster formation which includes the CH selection phase.

Note that both the intra-cluster and inter-cluster communications in single-hop clustering routing protocols require no form of intermediate nodes, as in the case of the multi-hop clustering routing protocols. Therefore, the cluster formation stages should be carefully designed in order to evenly distribute the network load, so that the network lifetime and functionality are extended for a reasonable period. As a result, our classification of the decision variables used in conveying the sensed information to the BS in single-hop clustering routing protocols is limited to the cluster formation stages. That is, we grouped the studied single-hop clustering routing protocols based on how the CH is selected and how the nCH nodes choose the cluster to join. The data transmission phase requires no further design since all the nCH nodes transmit their information to their respective CH nodes directly. Likewise, the CH nodes transmit the aggregated data to the BS directly. Accordingly, the studied energy-efficient and energy-balanced single-hop clustering routing protocols are classified based on the cluster formation stages only, as shown in Table 6. In a similar format to Table 2, in Table 6, we firstly classified the studied energy-efficient and energy-balanced single-hop clustering routing protocols based on their network infrastructure as homogeneous and/or heterogeneous WSN. Subsequently, we grouped the various single-hop clustering routing protocols based on the choice of decision variable(s) used in selecting the CH. As represented in Table 6, the decision variables used by these single-hop clustering routing protocols to select the CH are grouped under “RE“, “distance”, “AE”, “RSS/TP“ and “predefined”. As explained in Section 5.1.1, these parameters have their pros and cons when used in selecting the CH nodes. Thus, researchers attempt to find a balance in using two or more of these decision variables. Moreover, we grouped these routing protocols that rotate the CH duty in order to avoid early network partitioning. After the CH selection phase, we grouped the routing protocols based on the approach in which the nCH nodes join a cluster. This phase of the cluster formation is equally important in order to evenly distribute the network load. Therefore, the decision variables used by these routing protocols to specify how the nCH nodes join a cluster are grouped under “RE”, “distance”, “RSS/TP”. Also, some authors [190] grouped the SNs based on their location. In doing this, they assume that the SNs are perfectly aware of their location. Although assuming that the SNs are aware of their location might not be practical in reality, we grouped the routing protocols that made this assumption in forming their cluster. In addition, in a similar approach as in Section 5.1.1, we classified the studied energy-efficient and energy-balanced single-hop clustering routing protocols based on the clustering formation into equal or unequal cluster size. Lastly, in Table 6, we strived to classify the single-hop clustering routing protocols based on the optimization solution types or algorithms.

## 6. Survey Findings and Possible Research Directions

Different research works are ongoing in order to proffer a reliable solution to the energy consumption optimization problem relating to WSNs. The choice of the design input decision variable or the combinations of the design input decision variables is still an open area of research. We emphasised that using a single decision variable such as the residual energy cannot guarantee an energy-efficient and energy-balanced route in most cases. As discussed, all these decision variables have their advantages and disadvantages. Therefore, efficiently integrating these design decision variables can proffer a better solution to the energy consumption issue relating to WSNs in comparison to using a single decision variable. However, efficiently integrating these design decision variables in order to always ensure an optimal energy-efficient and energy-balanced route towards the BS from the source node can be an NP-hard problem. Besides the trade-offs in combining these decision variables, the proposed mathematical formulation might exhibit a high computational time and complexity delay. Moreover, the choice of the input decision variables can be application specific. Thus, the developers of WSN routing protocols should consider the network requirements before selecting the input decision variables.

Furthermore, we observed that more than 50% of researchers use the residual energy, hop-distance, and hop count as the decision variables in deriving the routing protocol mathematical formulation. With the residual energy, they ensure that the participating SN has sufficient energy to receive and convey the sensed information for that particular data transmission round. The hop-distance which is a very important parameter in the WSN energy dissipation model is used to choose the next hop. The smaller the hop-distance, the less energy consumed in conveying the message. Hence, the importance of a small hop-distance. Lastly, the hop count is used to specify the number of relay node chains to the BS, especially in multi-hop communication methods. It is desirable to keep the hop count as small as possible in order to reduce the energy consumed in receiving and forwarding the message. Additionally, as shown in Table 2, Table 3, Table 4, Table 5 and Table 6, other parameters such as the AE and RSS/TP, can also be integrated in deriving the mathematical formulations while developing an energy-efficient and energy-balanced routing protocol. However, from the study of the WSN energy dissipation model in Section 4.1, we observed that the sensed information or the *q* bit message forms an important part of the model (refer to Equations (4) and (6)). In fact, the higher the value of the *q* bit message, the more the energy consumed in receiving and forwarding the sensed message. The weight of the *q* bit message has been largely ignored by most researchers in designing their routing protocol. The knowledge of the *q* bit message can also serve as an important decision variable that can be integrated with other decision variables in order to design an energy-efficient and energy-balanced routing protocol. Logically, if the weight of the *q* bit message to be transmitted is known, and the residual energy of all the SNs in the network is known, we can easily determine the SNs that can conveniently forward the message towards the BS or form the set of relay node chains toward the BS. More importantly, the weight of the *q* bit message will be useful in designing a load-balanced multipath routing protocol. If the weight of the sensed information is known, the load can easily be shared among the selected paths, where the paths with a high energy level carry more load in comparison to the paths with a moderate energy level. Thus, the weight of the *q* bit message can be considered in future works as a decision variable in designing an optimal load-balanced routing protocol.

We also observed that the position of the BS plays an important role in the design of an energy-efficient and energy-balanced routing protocol. Although the position of the BS can be application specific, there should be a unified position for the BS based on each sensor network application. As shown in the WSN representation of Figure 1, Figure 2, Figure 4, Figure 7, Figure 8, Figure 9 and Figure 10, we assumed different positions for the BS. Likewise, in the studied routing protocols, the authors assumed different convenient positions for the BS. In fact, if the position of the BS is changed in some of the algorithms, the algorithms will not perform as expected. This means that such algorithms are dependent on the position of the BS. Even so, some algorithms [139,141] assume that the BS changes position in order to prevent the energy-hole phenomenon. Whereas, some algorithms assume that the BS is stationed at the centre of the sensor field, others assume that the BS is situated at a corner of the sensor field. Thus, the position of the BS is very important in designing a load-balanced routing protocol. The assumptions of the position of the BS by different researchers is quite conflicting. As previously mentioned, let us assume that the position of the BS is application specific; each application should have a unified position for the BS in order to verify the performance of any proposed routing protocol. We admit that researches are ongoing on node placement optimization problems relating to WSN. However, we emphasise that more research should focus on finding an optimal position for the BS. This is because, no matter how properly we place the SNs, if the BS is poorly cited, the SNs will deplete their battery energy quickly during data transmission. Besides, the energy-hole problem might be more pronounced in such a network. Additionally, determining an optimal unified position for the BS will create a suitable platform to verify the performance of any proposed energy-efficient and energy-balanced routing protocol.

Lastly, in this paper, we presented a detailed survey on the common input decision variables used by researchers to provide a formalism of mathematical optimization structure for the routing problem relating to an energy-efficient and energy-balanced WSN. As previously mentioned, optimization plays a vital role in WSNs. A detailed optimization problem consists of the input decision variables, constraints, objective functions and the outputs. Therefore, future researches can provide a general formalism of mathematical optimization structure for the routing problem relating to an energy-efficient and energy-balanced WSN. In this case, similar tables (Table 2, Table 3, Table 4, Table 5 and Table 6) can be developed for the constraints and objective functions. With such tables, a unifying and formal mathematical optimization structure can be developed for any class of routing protocol for a WSN based on the use of energy-efficient and energy-balanced criteria.

## 7. Conclusions

Energy consumption is a major optimization problem relating to WSN applications in general. Since SNs are mainly battery powered devices, the rate of energy consumption of these sensor devices must be properly managed in order to protract the network lifetime and functionality for a reasonable period. Managing the energy consumed by these SNs can be quite a challenging problem because of the network structure and formulations. However, researchers and practitioners have proposed various methods of managing the energy consumption in WSNs, during either data transmission or by the SN hardware devices. Concentrating on the energy consumed during data transmission, this survey paper reviewed different energy-efficient and energy-balanced routing protocols that attempt to extend the network lifetime and functionality by minimising the energy consumption in the network. Firstly, in Section 2, we summarised different survey papers on routing protocols for WSNs and explained the difference of these existing survey papers from our survey work. In subsequent sections, we emphasised the importance of an energy-efficient and energy-balanced routing protocol, and introduced a taxonomy to classify the studied routing protocols based on their proposed mode of communication towards the BS. In explaining the proposed taxonomy, we grouped the studied routing protocols based on the design decision variables used in selecting the path(s) to BS. We established the strengths and weaknesses of the choice of the decision variables used in the design of these energy-efficient and energy-balanced routing protocols. Additionally, we classified these routing protocols based on the solution types or algorithms used to solve this energy consumption optimization problem. Thus, keeping in view the coverage of this survey paper relating to energy consumption optimization problems, we envisage that this paper will open up possible research directions in developing an energy-efficient and energy-balanced routing protocol.

## Figures and Tables

**Table 1:** Summary of related works.

Year	Survey Paper	Contributions
2002	[30,31]	✓ Discussed the potential applications and factors affecting the design of WSNs. ✓ Outlined the communication architecture for WSNs.
2004	[11]	✓ Analysed the difficulties in designing a routing protocol for WSNs. ✓ Classified routing strategies into flat, hierarchical, and location-based routing. ✓ Defined some metrics such as negotiation-based, QoS-based, multipath-based to classify routing protocols. ✓ Compared the different routing protocols for WSNs, stating their strengths and weaknesses.
2005	[32]	✓ Provided a general survey on routing protocols for WSNs. ✓ Classified routing protocols into data-centric, hierarchical, and location-based.
2006	[12]	✓ Discussed the challenges and logic in developing a clustering algorithm for WSNs. ✓ Discussed the problems that face the practical design of clustering routing techniques for WSN applications. ✓ Classified clustering routing algorithms for WSNs based on the clustering objectives and design principles.
2007	[13]	✓ Introduced a taxonomy to classify clustering routing protocol. ✓ Discussed the strengths and weaknesses of different clustering routing algorithms. ✓ Compared different clustering techniques using some measures such as cluster stability, location-awareness, convergence rate.
2008	[33]	✓ Provided an overview of different WSN applications. ✓ Classified the challenges in WSNs into three categories. ✓ Presented the main research development in the mentioned categories.
2009	[34]	✓ Provided a survey on energy consumed by SN hardware components. ✓ Divided SN’s hardware into four main components. ✓ Classified the energy saving schemes for WSNs into duty-cycling, data-driven, and mobility-based. ✓ Elaborated on the importance of conserving energy consumed by SN hardware components.
2010	[38]	✓ Discussed and compared different energy-efficient hierarchical cluster-based routing protocols for WSNs.
2011	[35]	✓ Provided a survey on energy-efficient routing protocols for WMSNs. ✓ Defined the design challenges and limitations of energy-efficient routing protocols for WMSNs. ✓ Classified the energy-efficient routing protocols for WMSNs based on some metrics such as QoS requirement, data delivery model.
2011	[36]	✓ Provided a survey on swarm intelligence-based routing protocols for WSNs. ✓ Discussed the general principles and applications of swarm intelligence-based routing for WSNs. ✓ Proposed a taxonomy to classify swarm intelligence-based routing protocols for WSNs.
2011	[14]	✓ Provided a survey on energy-efficient clustering routing protocols for heterogeneous WSNs. ✓ Compared fifteen routing protocols based on the clustering method, clustering attributes, location-awareness, and heterogeneity level.
2012	[39]	✓ Classified clustering routing protocols based on their objectives and methods. ✓ Provided a taxonomy to the studied clustering routing protocols. ✓ Discussed the strengths and weaknesses of the techniques used in the studied clustering routing protocols. ✓ Summarises the issues and solutions of the attributes and characteristics of clustering approaches.
2012	[15]	✓ Presented the advantages and applications of clustering techniques for WSNs. ✓ Introduced a taxonomy to classify clustering routing protocols for WSNs. ✓ Compared different clustering routing protocols based on measures such as scalability, energy efficiency, cluster stability, load balancing.
2012	[37]	✓ Classified energy-efficient routing protocols into three categories, stating their strengths and weaknesses. ✓ Explained the areas of application of different energy-efficient routing protocols.
2012	[16]	✓ Discussed the notion and challenges of multipath routing protocols for WSNs. ✓ Classified the surveyed multipath routing protocols while outlining their pros and cons. ✓ Summarised the surveyed multipath routing protocols based on their network applications.
2012	[17]	✓ Classified multipath routing protocols for WSNs into infrastructure-based, non-infrastructure-based, and coding-based. ✓ Explained the evaluation metric, objectives, and challenges in designing a multipath routing protocols for WSNs.
2013	[18]	✓ Investigated the advantages of different multipath routing protocols for WSNs. ✓ Classified different multipath routing protocols for WSNs based on their features.
2013	[4]	✓ Provided a survey on energy-efficient routing protocols for WSNs. ✓ Classified energy-efficient routing protocols for WSNs based on the topology, communication model, network structure, and reliable routing schemes ✓ Compared different energy-efficient routing protocols for WSNs, stating their advantages and disadvantages.
2014	[10]	✓ Provided a general overview of WSNs, stating the areas of application and challenges of WSNs. ✓ Reviewed the prime research work and testbeds, standards and platforms, and the techniques and principles of WSNs. ✓ Outlined the current happenings in WSN research that considers the possible interaction between WSNs and other technologies.
2016	[19]	✓ Compared different multipath routing protocols for WMSNs based on their working operations. ✓ Provided the advantages and disadvantages of different multipath routing protocols for WMSNs.

**Table 2:** Decision variables for CH selection and formation in a multi-hop clustering network.

Year	Clustering Routing Protocol	HM	HT	CH Rotation	Decision Variables (CH Selection)						LA	Decision Variables (Cluster Formation)				
					RE	DBS	HCBS	TB	TPBS	Predefined		RE	DCH	HCCH	TPCH	Others
2000	[89]	✓		✓			✓							✓		
2002	[101]	✓	✓						✓						✓	
2003	[102]	✓		✓	✓	✓	✓						✓			
2003	[55]		✓		✓						✓		✓			
2003	[103]		✓							✓					✓	
2004	[56]	✓	✓	✓	✓								✓	✓		
2004	[83]	✓		✓		✓							✓			
2004	[104]	✓	✓	✓	✓								✓	✓		
2005	[105]	✓	✓							✓	✓		✓			
2005	[90]	✓		✓	✓			✓							✓	
2005	[57]	✓		✓	✓								✓			
2005	[58]	✓		✓	✓								✓			
2005	[94]	✓									✓					
2006	[84]	✓		✓		✓							✓			
2006	[106]	✓		✓	✓		✓						✓			
2006	[107]	✓		✓							✓		✓			
2006	[108]	✓		✓	✓		✓						✓			
2007	[109]	✓		✓		✓				✓	✓				✓	
2008	[110]		✓							✓	✓					
2008	[95]		✓							✓			✓			
2008	[99]		✓							✓	✓					
2008	[111]		✓							✓			✓			
2008	[112]	✓		✓	✓	✓							✓			
2008	[113]	✓		✓	✓					✓	✓		✓			
2008	[85]	✓		✓		✓							✓			
2008	[114]		✓							✓	✓					
2008	[59]	✓		✓	✓									✓		
2008	[60]	✓		✓	✓						✓					
2008	[61]	✓		✓	✓							✓	✓			
2009	[62]	✓		✓	✓						✓		✓			
2009	[115]	✓		✓	✓	✓									✓	
2009	[63]	✓		✓	✓							✓				
2009	[96]		✓							✓			✓			
2009	[64]	✓		✓	✓										✓	
2009	[116]	✓		✓						✓					✓	
2009	[117]		✓	✓	✓	✓							✓			
2010	[118]	✓		✓	✓		✓							✓		
2010	[119]	✓		✓	✓	✓							✓			
2010	[120]		✓	✓	✓	✓			✓		✓	✓	✓			
2010	[65]	✓		✓	✓						✓			✓		
2011	[66]		✓	✓	✓									✓		
2011	[67]	✓		✓	✓							✓	✓			
2011	[91]	✓		✓	✓			✓			✓				✓	
2011	[121]	✓		✓	✓	✓							✓			
2011	[122]	✓		✓	✓	✓								✓		
2011	[68]		✓	✓	✓								✓			
2012	[69]		✓	✓	✓								✓			
2012	[123]	✓		✓	✓				✓		✓		✓			
2012	[86]	✓		✓		✓							✓			
2012	[70]	✓		✓	✓						✓		✓			
2012	[71]	✓		✓	✓									✓		
2012	[124]		✓							✓	✓				✓	
2012	[125]		✓							✓	✓		✓			
2012	[72]	✓		✓	✓							✓				
2012	[97]		✓							✓		✓	✓			
2012	[98]		✓							✓						✓
2012	[87]	✓		✓		✓							✓			
2012	[73]	✓		✓	✓								✓			
2013	[74]	✓		✓	✓						✓		✓			
2013	[75]	✓		✓	✓						✓		✓		✓	
2013	[126]		✓							✓					✓	
2013	[127]		✓							✓			✓			
2013	[92]	✓		✓	✓			✓					✓			
2013	[128]		✓	✓	✓	✓							✓			
2013	[129]	✓		✓	✓	✓					✓	✓	✓			
2013	[130]	✓		✓	✓	✓							✓			
2014	[131]		✓							✓		✓	✓			
2014	[76]	✓		✓	✓								✓			
2014	[88]		✓	✓		✓								✓		
2014	[77]	✓		✓	✓								✓			
2014	[41]		✓							✓			✓	✓		
2015	[132]	✓		✓	✓				✓		✓	✓				
2015	[133]	✓		✓	✓	✓					✓				✓	
2015	[78]	✓		✓	✓							✓	✓			
2015	[79]	✓		✓	✓							✓				
2015	[93]	✓		✓				✓					✓			
2015	[80]	✓		✓	✓								✓			
2015	[134]	✓		✓	✓	✓							✓			
2015	[135]	✓		✓	✓		✓					✓	✓			
2015	[81]	✓		✓	✓										✓	
2015	[136]	✓		✓				✓					✓	✓		
2015	[137]	✓		✓	✓								✓			
2016	[138]	✓		✓	✓	✓							✓			
2016	[139]		✓	✓	✓	✓										✓
2016	[82]	✓		✓	✓						✓	✓				

**Table 3:** Decision variables for multi-hop routing towards the BS.

Year	Clustering Routing Protocol	Cluster Only	Intra-Cluster Communication (Decision Variables)					Inter-Cluster Communication (Decision Variables)					CHtoBS		Cluster Size		Optimization Approach
			Direct	RE	Hop-Distance	Next Hop	Others	Direct	RE	Hop-Distance	Next Hop	Others	CH	nCH	Equal	Unequal	
2000	[89]	✓													✓		H
2002	[101]	✓													✓		H
2003	[102]	✓													✓		S
2003	[55]		✓	✓	✓		✓	✓		✓			✓		✓		H
2003	[103]		✓	✓	✓		✓	✓		✓			✓		✓		H
2004	[56]		✓		✓			✓		✓			✓		✓		H
2004	[83]	✓													✓		H
2004	[104]		✓		✓			✓		✓			✓		✓		H
2005	[105]		✓							✓			✓			✓	H
2005	[90]		✓							✓			✓		✓		H
2005	[57]	✓													✓		H
2005	[58]		✓							✓			✓		✓		H
2005	[94]		✓		✓			✓		✓			✓	✓	✓		H
2006	[84]		✓		✓			✓		✓			✓		✓		H
2006	[106]		✓		✓			✓			✓	✓	✓		✓		S
2006	[107]	✓													✓		H
2006	[108]		✓					✓			✓		✓		✓		H
2007	[109]		✓					✓		✓			✓		✓		H
2008	[110]		✓		✓			✓		✓			✓		✓		L
2008	[95]		✓	✓	✓		✓	✓							✓		L
2008	[99]						✓	✓							✓		H
2008	[111]		✓		✓	✓		✓		✓	✓		✓		✓		L
2008	[112]		✓					✓		✓			✓		✓		H
2008	[113]		✓		✓			✓		✓			✓		✓		S
2008	[85]		✓		✓			✓		✓			✓		✓		H
2008	[114]		✓		✓		✓	✓				✓	✓		✓		G
2008	[59]		✓					✓		✓			✓		✓		H
2008	[60]		✓					✓	✓				✓			✓	H
2008	[61]		✓							✓			✓			✓	H
2009	[62]		✓							✓		✓	✓			✓	H
2009	[115]		✓					✓	✓	✓			✓			✓	H
2009	[63]		✓					✓			✓		✓			✓	H
2009	[96]		✓	✓		✓		✓							✓		H
2009	[64]	✓													✓		H
2009	[116]		✓					✓	✓				✓		✓		H
2009	[117]		✓					✓	✓	✓		✓	✓		✓		H
2010	[118]		✓								✓		✓		✓		H
2010	[119]	✓													✓		H
2010	[120]		✓	✓	✓			✓							✓		H
2010	[65]	✓													✓		H
2011	[66]		✓					✓	✓		✓		✓		✓		H
2011	[67]		✓					✓				✓	✓		✓		H
2011	[91]		✓				✓	✓				✓	✓	✓	✓		H
2011	[121]		✓					✓			✓		✓		✓		H
2011	[122]		✓					✓			✓		✓		✓		H
2011	[68]		✓					✓			✓		✓			✓	H
2012	[69]		✓					✓			✓		✓		✓		H
2012	[123]		✓					✓			✓		✓		✓		H
2012	[86]		✓					✓			✓		✓		✓		H
2012	[70]		✓					✓			✓		✓		✓		H
2012	[71]		✓					✓			✓		✓			✓	H
2012	[124]		✓					✓	✓		✓	✓	✓		✓		H
2012	[125]		✓					✓			✓		✓		✓		H
2012	[72]		✓					✓			✓		✓			✓	H
2012	[97]		✓	✓	✓			✓							✓		S
2012	[98]		✓					✓				✓	✓		✓		H
2012	[87]		✓					✓			✓		✓		✓		H
2012	[73]		✓					✓	✓	✓		✓	✓		✓		H
2013	[74]		✓					✓			✓		✓		✓		S
2013	[75]		✓					✓	✓				✓		✓		H
2013	[126]		✓			✓		✓							✓		H
2013	[127]		✓					✓			✓		✓		✓	✓	E
2013	[92]		✓					✓			✓		✓		✓		B
2013	[128]		✓			✓		✓	✓				✓		✓		H
2013	[129]		✓			✓		✓			✓		✓		✓		H
2013	[130]		✓			✓		✓			✓		✓			✓	H
2014	[131]		✓			✓		✓			✓		✓		✓	✓	L
2014	[76]		✓		✓			✓		✓			✓		✓		H
2014	[88]		✓					✓			✓		✓		✓		H
2014	[77]		✓					✓			✓		✓		✓		M
2014	[41]		✓					✓			✓		✓		✓		B
2015	[132]		✓	✓	✓			✓	✓	✓			✓		✓		H
2015	[133]		✓		✓			✓		✓			✓		✓		B
2015	[78]		✓					✓	✓		✓		✓		✓		H
2015	[79]		✓					✓			✓		✓		✓		H
2015	[93]	✓													✓		H
2015	[80]		✓					✓			✓		✓		✓		H
2015	[134]		✓					✓	✓	✓			✓		✓		E
2015	[135]		✓					✓	✓	✓		✓	✓		✓		H
2015	[81]		✓					✓				✓	✓	✓	✓		H
2015	[136]		✓			✓		✓			✓		✓	✓	✓		H, B
2015	[137]		✓					✓		✓			✓		✓		H
2016	[138]		✓					✓	✓				✓			✓	H
2016	[139]		✓			✓			✓						✓		H
2016	[82]		✓					✓		✓			✓		✓		G

**Table 4:** Decision variables for multipath load-balanced tree routing towards the BS.

Year	Multipath Routing Protocol	HM	HT	LA	Path Discovery					Path Selection/Load Distribution						Optimization Approach
					RE	HC	RSS/TP	Hop-Distance	Others	RE	HC	TE	Rate	Hop-Distance	Others	
2000	[143]	✓			✓	✓				✓	✓	✓				H
2002	[144]		✓		✓	✓				✓	✓					H
2004	[145]	✓			✓	✓				✓	✓	✓				H
2007	[146]	✓			✓	✓			✓	✓	✓				✓	H
2007	[147]	✓			✓	✓	✓						✓			H
2007	[148]	✓			✓	✓				✓	✓					H
2008	[149]	✓		✓		✓			✓		✓				✓	H
2008	[150]	✓			✓			✓							✓	H
2010	[151]	✓						✓	✓		✓				✓	H
2011	[152]	✓		✓	✓	✓			✓	✓	✓				✓	H
2012	[153]	✓			✓	✓	✓						✓			H
2012	[154]	✓				✓					✓					H
2014	[155]	✓				✓			✓	✓			✓		✓	H
2014	[156]	✓				✓			✓	✓			✓		✓	H
2015	[157]	✓				✓				✓				✓	✓	H
2015	[158]	✓		✓	✓					✓	✓		✓			H

**Table 5:** Decision variables for single-path load-balanced tree routing towards the BS.

Year	Single-Path Routing Protocol	HM	HT	LA	Single-Path Load Distribution (Decision Variables)							Optimization Approach
					RE	Hop-Distance	HC	TE	RSS/TP	Throughput	AE	
2001	[171]	✓					✓					H
2002	[166]	✓		✓	✓	✓						H
2003	[174]	✓				✓			✓			H
2005	[175]	✓	✓		✓							B
2006	[176]	✓			✓	✓					✓	H
2007	[177]	✓			✓		✓					H
2007	[178]	✓		✓	✓		✓		✓			H
2008	[168]	✓			✓	✓	✓					H
2009	[169]	✓		✓	✓	✓	✓					H
2009	[179]	✓			✓	✓					✓	H
2009	[180]	✓			✓	✓			✓			H
2010	[172]	✓		✓		✓						H
2010	[181]		✓		✓			✓				H
2011	[182]	✓			✓				✓			H
2011	[183]	✓			✓		✓					H
2011	[170]	✓			✓	✓	✓					H
2013	[184]	✓				✓						H
2013	[185]	✓		✓		✓						H
2014	[167]	✓			✓	✓						H
2015	[186]	✓				✓						H
2016	[173]	✓								✓		H

**Table 6:** Decision variables for CH selection and formation in a single-hop clustering network.

Year	Clustering Routing Protocol	HM	HT	CH Rotation	Decision Variables (CH Selection)					LA	Decision Variables (Cluster Formation)			Cluster Size		Optimization Approach
					RE	Distance	AE	RSS/TP	Predefined		RE	Distance	RSS/TP	Equal	Unequal	
2002	[50]	✓		✓					✓			✓		✓		H
2005	[187]	✓		✓					✓	✓		✓		✓		H
2005	[188]	✓		✓	✓							✓		✓		H
2006	[189]		✓	✓	✓			✓				✓		✓		H
2007	[190]		✓	✓	✓			✓		✓				✓		H
2008	[191]	✓		✓	✓	✓						✓		✓		H
2008	[192]		✓	✓	✓							✓		✓		H
2009	[193]	✓		✓	✓	✓						✓		✓		H
2009	[194]	✓		✓	✓								✓	✓		H
2011	[195]	✓		✓	✓		✓			✓	✓			✓		H
2011	[196]	✓	✓	✓	✓	✓						✓		✓		E
2012	[197]	✓		✓	✓					✓					✓	H
2012	[198]		✓						✓	✓		✓		✓		H
2012	[100]	✓		✓	✓		✓					✓		✓		H
2015	[199]		✓	✓	✓		✓			✓		✓			✓	G
2016	[200]	✓		✓	✓	✓						✓		✓		H
